# Robust Performance of Marginal Pacific Coral Reef Habitats in Future Climate Scenarios

**DOI:** 10.1371/journal.pone.0128875

**Published:** 2015-06-08

**Authors:** Lauren A. Freeman

**Affiliations:** NRC Postdoctoral Fellow, Remote Sensing Division, Naval Research Laboratory, Washington, D.C., United States of America; Centro de Investigacion Cientifica y Educacion Superior de Ensenada, MEXICO

## Abstract

Coral reef ecosystems are under dual threat from climate change. Increasing sea surface temperatures and thermal stress create environmental limits at low latitudes, and decreasing aragonite saturation state creates environmental limits at high latitudes. This study examines the response of unique coral reef habitats to climate change in the remote Pacific, using the National Center for Atmospheric Research Community Earth System Model version 1 alongside the species distribution algorithm Maxent. Narrow ranges of physico-chemical variables are used to define unique coral habitats and their performance is tested in future climate scenarios. General loss of coral reef habitat is expected in future climate scenarios and has been shown in previous studies. This study found exactly that for most of the predominant physico-chemical environments. However, certain coral reef habitats considered marginal today at high latitude, along the equator and in the eastern tropical Pacific were found to be quite robust in climate change scenarios. Furthermore, an environmental coral reef refuge previously identified in the central south Pacific near French Polynesia was further reinforced. Studying the response of specific habitats showed that the prevailing conditions of this refuge during the 20^th^ century shift to a new set of conditions, more characteristic of higher latitude coral reefs in the 20^th^ century, in future climate scenarios projected to 2100.

## Background

Coral reef ecosystems are among the most biodiverse on earth, described as “rainforests of the sea” [1]. They provide many ecosystem services, including coastal protection, recreation and tourism, new substances used by the pharmaceutical industry, and natural beauty [2–3]. Tens of millions of people depend on coral reefs for protein and livelihood in coastal nations [4]. The ability of coral reefs to persist in the next century is under threat from both climate change and human stress. Human stressors include artisanal and commercial fishing pressure, nutrient runoff, and pollution [5–10]. However, coral reefs face an even greater threat from climate change. Two consequences of rising atmospheric carbon dioxide (CO_2_) have been well documented in terms of their adverse effects on coral reefs–higher ocean temperatures and lower aragonite saturation state [11–15].

Previously coral reefs have been considered to exist in a fairly uniform physico-chemical environment, but recent work has shown that coral reefs in fact occupy a wide range of environments [16–18]. These environments are characterized by varying sea surface temperatures, nutrient levels, seasonal variability, and other physico-chemical parameters. Some of the environments are near the fringe of ‘acceptable’ conditions for coral reefs, known as “marginal habitats” [19]. These marginal habitats are often considered most at risk from climate change.

### Environmental Variables Affecting Coral Reef Ecosystems

Increased CO_2_ in seawater results in decreased pH (ocean acidification) and an associated **decrease in aragonite saturation state**. The reduced aragonite saturation state reduces the ability of corals to calcify and build skeletons and reef structure [12,15,20–21]. At some point, acidification will cause existing reefs to erode faster than corals can deposit more structure. Aragonite saturation state is a key indicator of the ability of corals to build structure. Because CO_2_ dissolves more readily in cooler water, decreasing aragonite saturation state is a more serious concern at higher latitudes, limiting coral reef habitation poleward [13,19].

**Fig 1:**
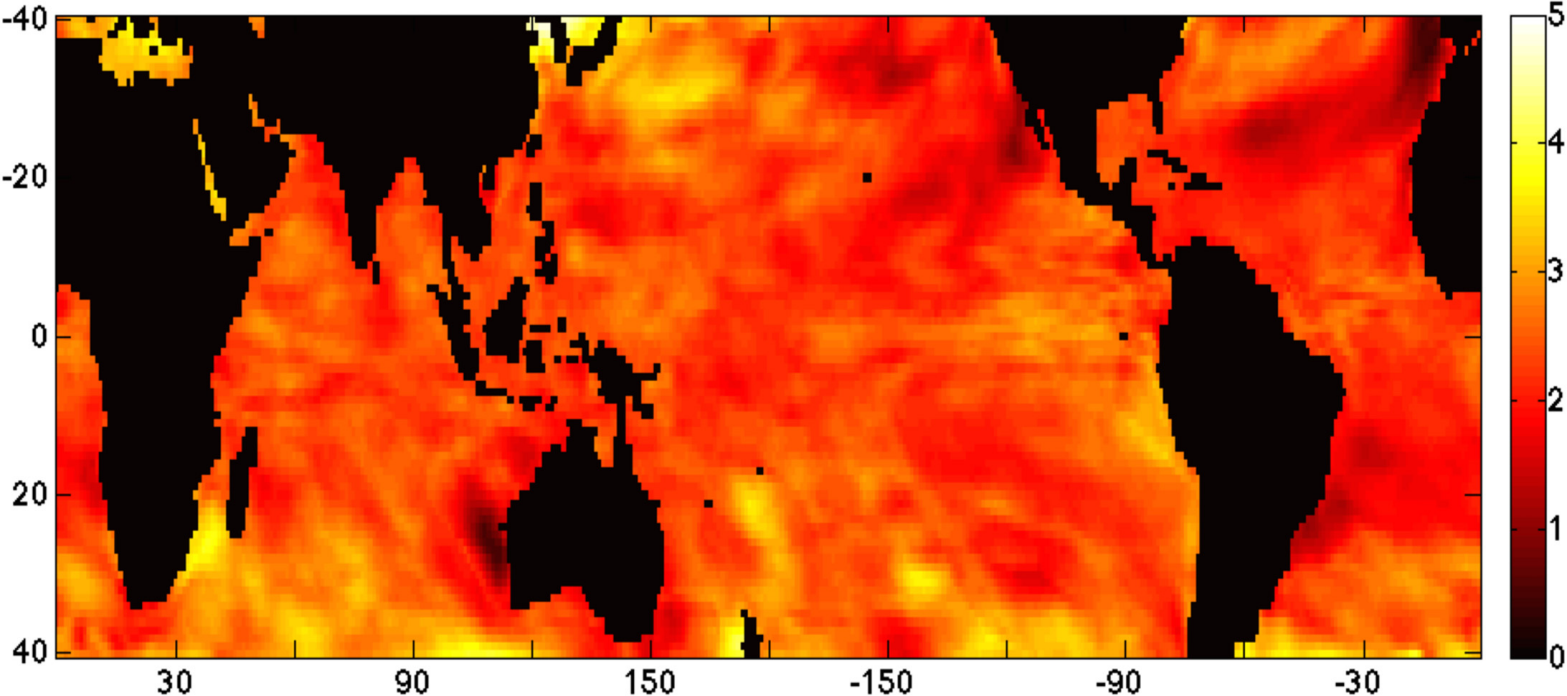
Map of Spatial Heterogeneity in Sea Surface Temperature Change from 2010–2100. The difference in average maximum sea surface temperatures from time period 2006–2010 to 2096–2100 is shown in the above map, calculated from five-year averages from the NCAR CESM1 RCP8.5 scenario. Scale indicates magnitude of change from low (dark red) to high (white) in °C. Landmasses are black.

**Rising sea surface temperatures** result in a breakdown of coral-algae symbiosis in which the dinoflagellates are ejected from the coral (coral bleaching), often resulting in disease or mortality to the coral [11,21–22]. The increase in maximum tropical SSTs will be between 0.4–4.8°C between the years 2010–2100 for the business-as-usual scenario in the National Center for Atmospheric Research (NCAR) Community Earth System Model (CESM1). This change is distributed non-uniformly around the world (Fig 1). Changes in mean temperature are accompanies by shifts in temperature variability and temperature anomalies. Regions that previously experienced static conditions year-round may be subject to large swings in ocean temperature. Of particular importance for coral reefs are high temperature anomalies. These extremes are often quantified as **degree heating weeks** (DHW), which are calculated as a rolling summation of temperatures that exceed the climatological mean by more than one degree Celsius. Temperature anomalies such as DHWs are correlated with coral bleaching [23].

Rising temperature tends to be the most commonly studied aspect of how climate change impacts marine ecosystems, and ocean acidification is increasingly recognized for its potential to cause a range of impacts on marine organisms and ecosystems [24]. However, many other aspects of basic oceanic habitats will also shift. **Ocean current patterns** will change, resulting in the dislocation of upwelling zones and transport pathways. Near landmasses, rainfall will alter terrestrial runoff frequency and patterns, affecting **nutrient levels** for coastal ocean ecosystems. **Atmospheric cyclones** are projected to increase in intensity and possibly frequency [25–27], further affecting the shallow seas. The geographical limits of shallow-water coral reef ecosystems are also defined by **salinity** and light availability as **photosynthetically active radiation (PAR)**, [17,19], and other variables that are more difficult to quantify such as species interactions and connectivity.

When oceanographic conditions change synergistically, the result is a related shift in the geographic range of suitable ocean habitat suitable for particular ecosystems, such as coral reefs. While tropical coral reefs all contain the same general ecological functional groups, they develop and exist across a large range of oceanic habitats [17–18]. The mean habitat suitability for coral reefs differs across the three tropical ocean basins (Caribbean, Pacific, and Indian), a reflection of unique environmental conditions in each [28]. There is further variation within each basin [16–17] and within specific regions [18]. While individual coral colonies are acclimatized to the conditions of their own unique location, each region will experience a different combination of environmental shifts associated with climate change.

### Ecosystem Niche Model

The ecosystem niche model Maxent [29] has been used in several studies to assess impacts of climate change on habitat suitability for ecosystems [28,30–31]. In the case of coral reefs, which span a diverse range of oceanic habitats, an important consideration in predictive studies is the set or subset of presence locations used for model training and calculation of habitat suitability [28]. Coral reefs occupy a wide range of physico-chemical conditions within [16–18] and between [28] ocean basins. Maxent calculates habitat suitability based on the range of physico-chemical conditions for the subset of locations given to the model for training. Thus, an effective use of Maxent for coral reefs may be to consider only coral reefs as they group into unique physico-chemical habitats.

### Objectives of This Study

There are two main objectives of the work presented here. 1: to generate future projections of coral reef habitat suitability for narrow ranges of environmental variables that describe unique coral habitats, building upon previous research [28] and 2: to test the utility of Maxent for generating these projections with relatively small sample sizes. To achieve the first objective, seven distinct coral reef habitats in the Pacific Ocean [17] are used as subsets for training Maxent to create future habitat suitability projections. The habitat suitability envelope results from each of the individual seven habitats are compared with results for the entire set of remote Pacific coral reefs. To realize the second objective, an analysis of sample size effects using random subsets of the same set of remote Pacific coral reefs is conducted. The assessment of sample size has far-reaching implications for other uses of Maxent, and also provides validity to the results for the individual coral reef habitats. A critical consideration in these studies is the importance of sample size for model training and calculation of habitat suitability.

## Methods

The ecosystem niche model Maxent is used to calculate coral reef habitat suitability for each of the seven unique physico-chemical habitats in the remote Pacific from Freeman et al. 2012. Habitat suitability is mapped in present and future climates using data from NCAR CESM1 earth system model. The methodology regarding CESM1, environmental variables, and Maxent follows that published in Freeman et al. 2013 for global coral reef analyses in present and future climates [28]. A series of tests are conducted with random samples of the remote Pacific coral reef dataset to determine the effect of sample size on Maxent calculations, validating the use of smaller training datasets in this study.

### Training Locations

**Fig 2:**
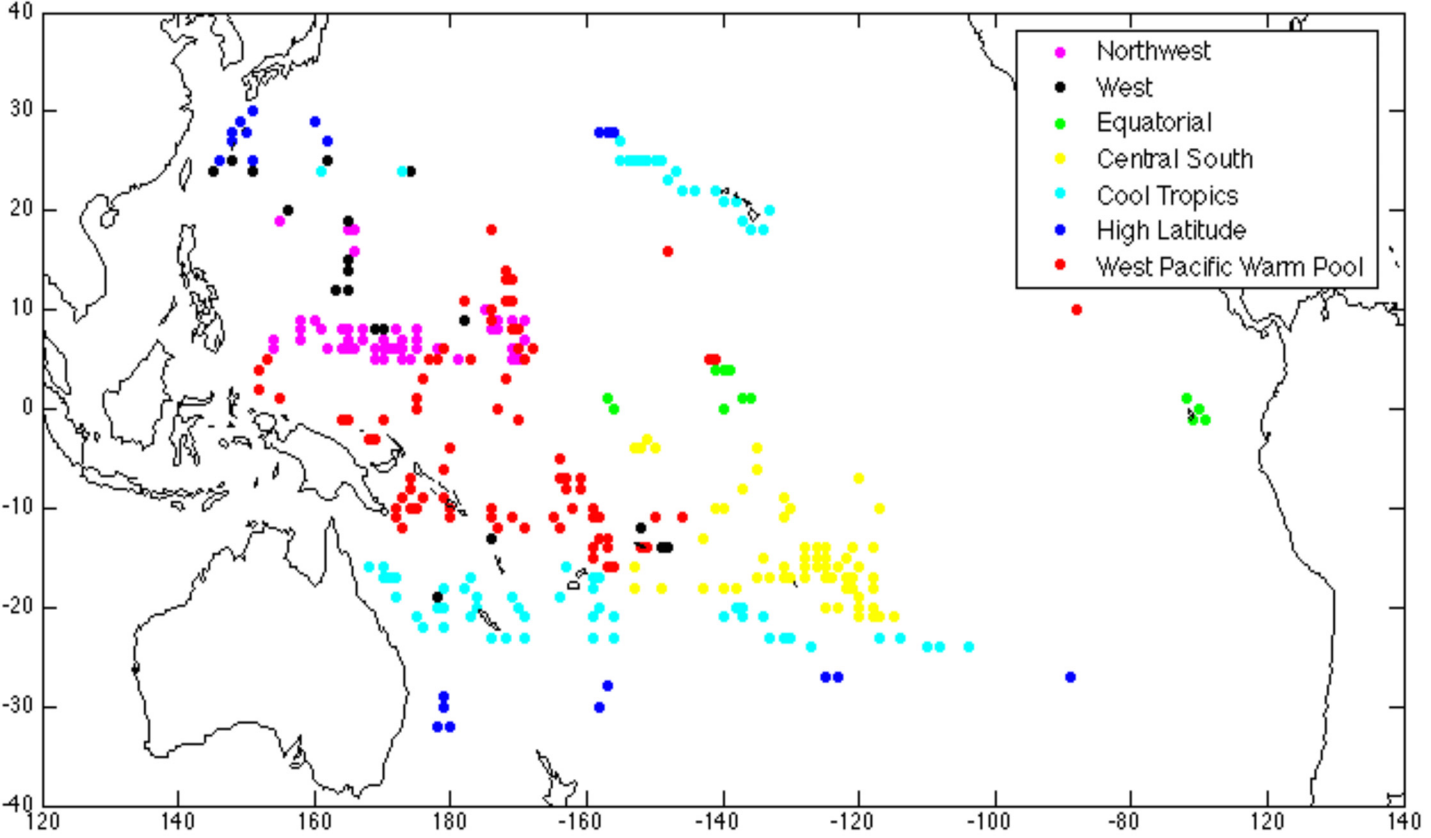
Map of Unique Physico-Chemical Coral Reef Habitats in the Remote Pacific. The seven unique physico-chemical coral reef habitats in the remote Pacific, mapped by color. Each colored circle identifies a remote coral reef location. The color indicates which habitat the coral reef falls into. Further description of these habitats can be found in Table 2. Copied with permission from Freeman et al. 2012 [17].

Coral-reef ecosystems presently exist in limited regions of the tropical and subtropical oceans. The coral reef locations (305 total locations, originally collected from ReefBase www.reefbase.org) used in this study were subdivided using a selection of physico-chemical variables (Table 1) with cluster analysis [17]. The resulting habitats are shown in Fig 2 and described in Table 2. In this study, the coordinates of coral reef locations within each habitat are used separately as training data to calculate habitat suitability with Maxent.

**Table 1:** Environmental variables used to determine unique coral reef habitats from 20^th^ century climatology.

Variable	Data Source	Notes	Type
Average sea surface temperature	World Ocean Atlas [50]	Cumulative average from early 1900s	Cruise data
Average sea surface phosphorus	World Ocean Atlas [51]	Cumulative average from early 1900s	Cruise data
Average sea surface dissolved oxygen	World Ocean Atlas [52]	Cumulative average from early 1900s	Cruise data
Average sea surface salinity	World Ocean Atlas [53]	Cumulative average from early 1900s	Cruise data
Average annual temperature variability	World Ocean Atlas [50]	Difference between warmest average month and coldest average month	Cruise data
Temperature anomaly- Degree Heating Weeks	NOAA Coral Reef Program	Average DHWs from 1985–2010	Satellite data
Average aragonite saturation state	GLODAP[54], WOA [50,53]	Calculated aragonite saturation state from 1985–2010	Satellite + cruise data
Annual storm frequency	NOAA Historic Hurricane Tracks	Includes all cyclonic storms within 110 km diameter cell	Satellite data + observations
Intense hurricane hits	NOAA Historic Hurricane Tracks	Category 4 and 5 hurricanes within 110 km diameter cell	Satellite data + observations

All variables were interpolated to 1 degree by 1 degree resolution and taken for specific locations in the remote Pacific containing coral reefs. Modified with permission from Freeman et al. 2012 [17].

**Table 2:** Description of Coral Reef Habitats.

Habitat	Annual Storm Frequency	Intense Hurricane Hits	Salinity (ppm)	Phosphate (μmol/kg)	Seasonal Temp^1^ Range (C)	Oxygen	Ω-arag^2^	DHW^3^ (C)	Mean Temp^1^ (C)
**Northwest Pacific**	0.62	0.00	34.23	0.13	0.91	4.54	3.95	0.19	28.90
**West Pacific**	0.61	1.11	34.67	0.12	2.84	4.60	3.91	0.31	27.74
**Equatorial Pacific**	0.00	0.00	34.86	0.55	1.84	4.63	3.59	1.62	26.31
**Central South Pacific**	0.04	0.00	35.84	0.29	1.78	4.63	4.15	0.17	27.63
**Cool Tropics**	0.14	0.00	35.29	0.14	3.51	4.76	3.85	0.27	25.50
**High Latitude Reefs**	0.28	0.00	35.14	0.12	5.92	4.93	3.60	0.46	23.37
**West Pacific Warm Pool**	0.13	0.00	34.60	0.20	1.10	4.55	3.87	0.22	28.70
**Northwest Pacific**	High	Avg^4^	Low	Low	Low	Low	Avg	Avg	High
**West Pacific**	High	Very High	Low	Low	Avg	Avg	Avg	Avg	Avg
**Equatorial Pacific**	Low	Avg	Avg	Very High	Avg	Avg	Low	Very High	Low
**Central South Pacific**	Low	Avg	High	High	Avg	Avg	High	Avg	Avg
**Cool Tropics**	Avg	Avg	Avg	Avg	High	High	Avg	Avg	Low
**High Latitude Reefs**	Avg	Avg	Avg	Low	Very High	Very High	Low	High	Very Low
**West Pacific Warm Pool**	Avg	Avg	Low	Avg	Low	Low	Avg	Avg	Avg

Numeric (top) and descriptive (bottom) values of environmental variables for each of the seven coral reef habitats in the remote Pacific. Modified with permission from Freeman et al. 2012 [17].

1:.Temperature

2: Aragonite Saturation State

3: Degree Heating Week

4: Average

### Sample Size Testing

In order to determine the effect of sample size, random selections of the original 305 location data set were taken at 10% intervals (10%, 20%, etc), to calculate habitat suitability using Maxent and compare with calculations and projections using the entire dataset. The 10% sample is comparable to the physico-chemical habitat with the least geographic coverage (Equatorial).

### Climate Model

The NCAR Community Earth System Model v1 (CESM1) is a global atmosphere-ocean, fully coupled climate model [32] that includes the Biogeochemical Element Cycle (BEC) model, containing information regarding nutrients and plankton [33]. The BEC model determines the complete suite of carbonate system components, which are used to estimate aragonite saturation state [34]. Environmental parameters were extracted from the CESM1 BEC 20^th^ century run (1985–2005) and two CESM1 BEC 21^st^ Century scenario runs described by unique representative carbon pathways (RCPs): RCP4.5 (2005–2100) and RCP8.5 (2005–2100). The BEC model was available for RCP4.5 and RCP8.5 only. RCP2.5 and RCP6.0 were not considered as a result. The two scenarios yield differences of approximately 2°C in mean global air temperature, with more warming for RCP8.5. Sea surface temperature, sea surface salinity, phosphate concentration at the sea surface (PO4), average light availability in the surface layer as PAR, surface horizontal by current velocities, and carbonate system parameter data were retrieved in a manner identical to that described in Freeman et al. 2013 [28].

### Environmental Variables and Application of Maxent

Environmental variables from CESM1 were used to train Maxent, a maximum entropy niche model that uses these variables combined with species presence data to determine the likelihood of suitable habitat for that species [29,35]. Previous studies show that Maxent can estimate geographical limits for a variety of marine and terrestrial ecological regimes in addition to those for individual species [36–40]. Some of these studies have demonstrated the ability of Maxent to accurately predict the distributions of present-day coral reefs [40]. Consequently, the application of climate model projections to Maxent to estimate the future geographic range of reefs using a “bioclimatic envelope” [41] is a natural step forward. A detailed description of the methods by which environmental variables extracted from the CESM1 climate model were applied to Maxent is found in Freeman et al. 2013 [28].

### Post-processing

All subsequent calculations were carried out in MATLAB Version 2012a including comparison of habitat suitability, percent change calculations, and statistics.

## Results

### Predictions For Remote Pacific Coral Reefs

**Fig 3:**
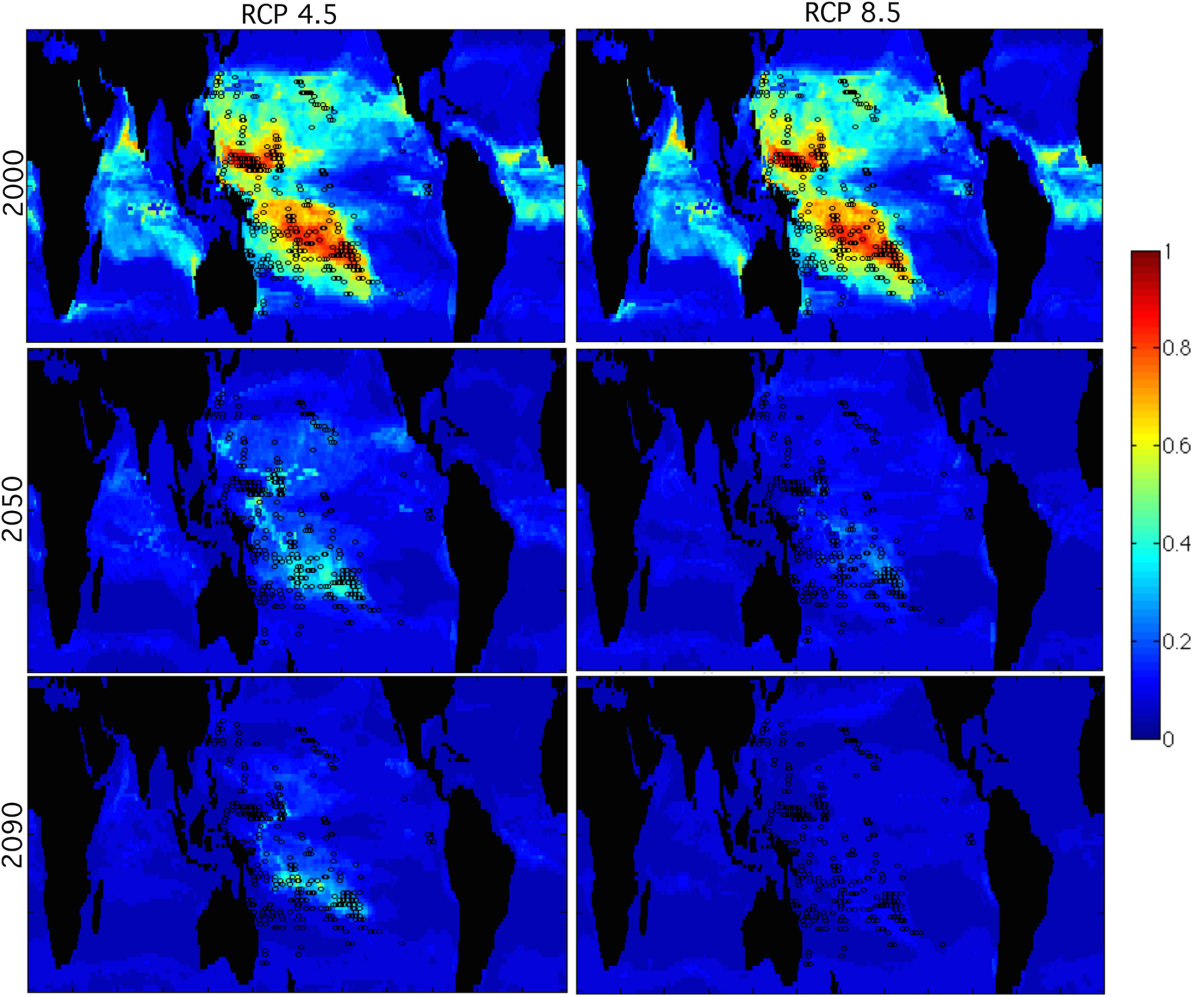
Maps Showing Suitable Habitat for All Remote Pacific Coral Reefs in Future Climate Projections. Maps of suitable habitat for all remote Pacific reefs in RCP4.5 (left column) and RCP8.5 (right column), shown for years 2000 (top), 2050 (middle), and 2090 (bottom). The color scale indicates the level of habitat suitability from marginal (blue) to highly suitable (yellow/red). Landmasses are shown in black. Coral reef locations used in this calculation are mapped as black circles.

When all remote Pacific reefs are used for Maxent training, general habitat constriction is found throughout 21^st^ century for both scenarios RCP4.5 and RCP8.5. Somewhat similar physico-chemical habitat (light blue in Fig 3) is available in most of the Pacific Ocean and parts of the Indian Ocean in 2100. This response is similar to previous habitat suitability for Pacific reefs [28] (Fig 3).

### Predictions By Oceanographic Habitat

**Fig 4:**
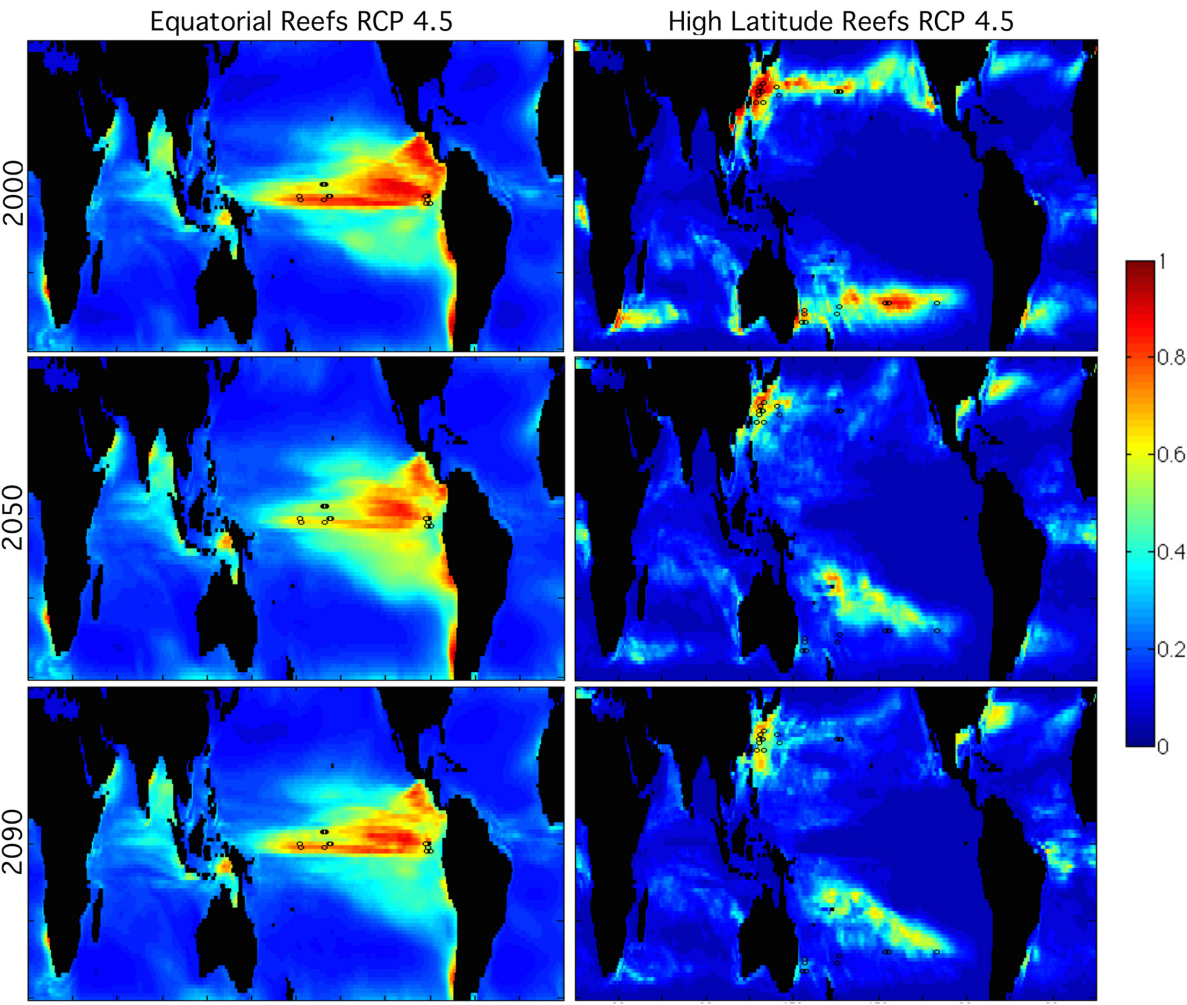
Maps Showing Robust Prevalence of Marginal Coral Reef Habitats in Future Climate Projections. Habitat suitability calculated from Maxent is shown as maps above for marginal reefs. These reefs show extended habitat in climate change scenarios as well as robust prevalence at original coral reef locations. Data from RCP4.5 is shown here. The Equatorial Reefs are in the left column and High Latitude Reefs are in the right column, shown for years 2000 (top), 2050 (middle), and 2090 (bottom). The color scale indicates the level of habitat suitability from marginal (blue) to highly suitable (yellow/red). Landmasses are shown in black. Coral reef locations used in this calculation are mapped as black circles.

Habitats with conditions that are currently considered as ‘marginal’ perform better than the habitats that currently offer the most ‘ideal’ conditions under the climate change projections presented here. By this we mean that previously described ‘marginal’ reefs [19] with physical or chemical environmental limitations have more persistent habitat in climate change scenarios than ‘ideal’ coral habitat in the western Pacific coral triangle region. Both the High Latitude and Equatorial reef regions retain extensive suitable habitat throughout the 21^st^ century in both RCP4.5 and RCP8.5 (Fig 4). Cooler regions subject to upwelling, including the eastern pacific, along the equator, and large portions of the Indian Ocean, remain relatively hospitable to reef building corals from the Eastern Tropical and Equatorial Pacific. Of the seven unique remote Pacific habitats tested here, High Latitude reefs retain the most extensive suitable habitat by the end of the 21^st^ century. The High Latitude Reef regions continue to indicate suitable habitat at higher latitudes, remain relatively suitable in the central south Pacific region, as well as in the Caribbean basin and along the Gulf Stream and Kuroshio Current (Fig 4).

When all remote Pacific reefs are considered for training, general habitat constriction is observed towards the end of the 21^st^ century, more severe for RCP8.5 (Fig 3). Cool Tropics, West, and West Pacific Warm Pool all show a similar trend. All of these cases highlight the South Pacific Refuge around French Polynesia [28]. Other physico-chemical habitats that are currently considered ‘ideal’ or ‘normal’ for coral reef ecosystems rapidly disappear, specifically the Northwest and Central South. Northwest show very little suitable habitat even in training, and rapidly have no suitable habitat, indicating that these are already marginal reefs.

### Effect of Sample Size on Predictions

**Fig 5:**
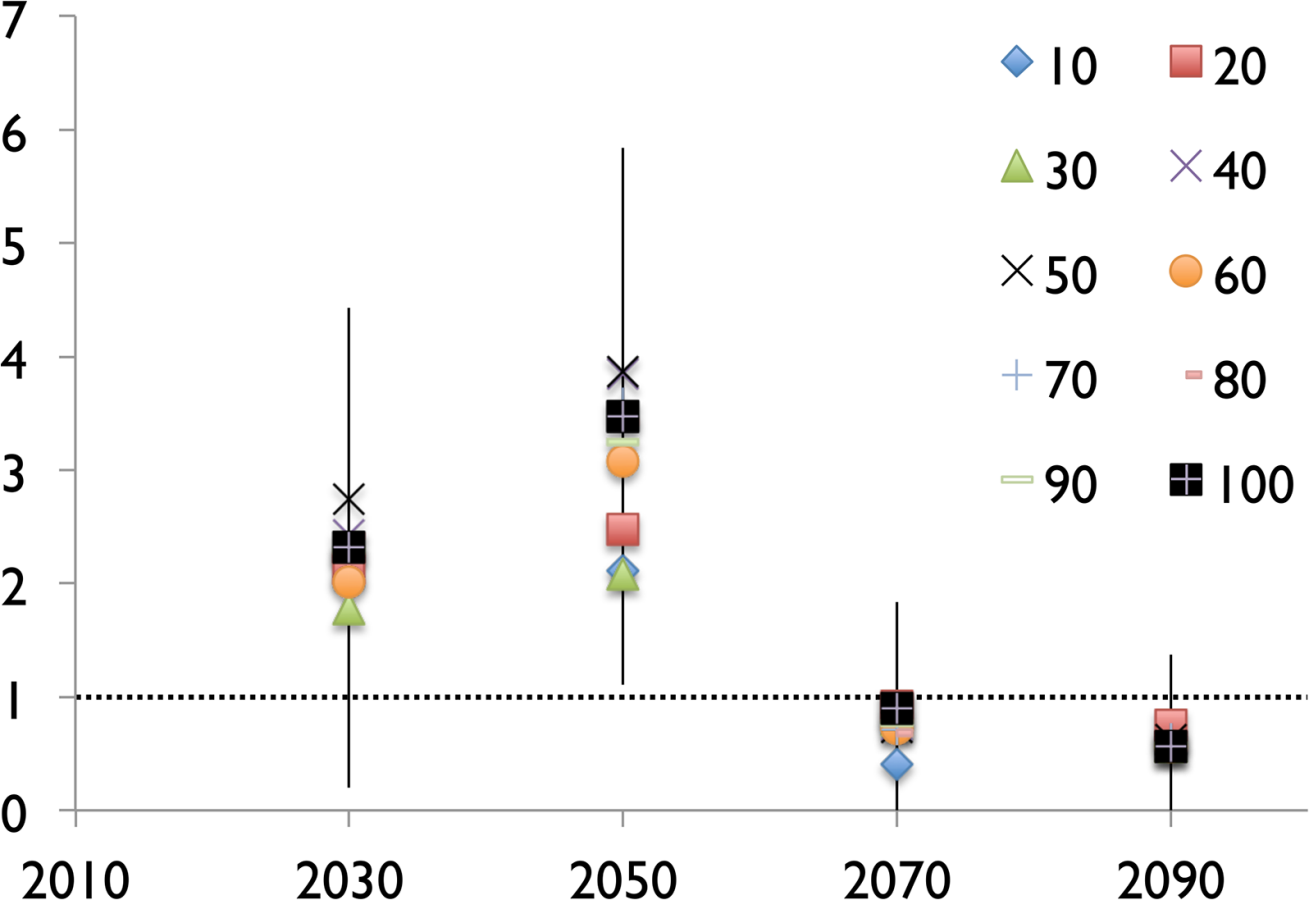
Effect of Maxent sample size on predictions. Scatterplot of mean habitat suitability index for 10–100% of remote Pacific locations used in training, plotted for 2030, 2050, 2070, and 2090. Error bars show 2x standard deviation of dataset using 100% of the potential locations for Maxent training. Dashed line indicates Maxent training value; plot is normalized to training values. The results show that the values obtained from as little as 10% of data points are well within the standard deviation of 100% of data points, giving Maxent results weight even for small sample sizes of training data.

**Fig 6:**
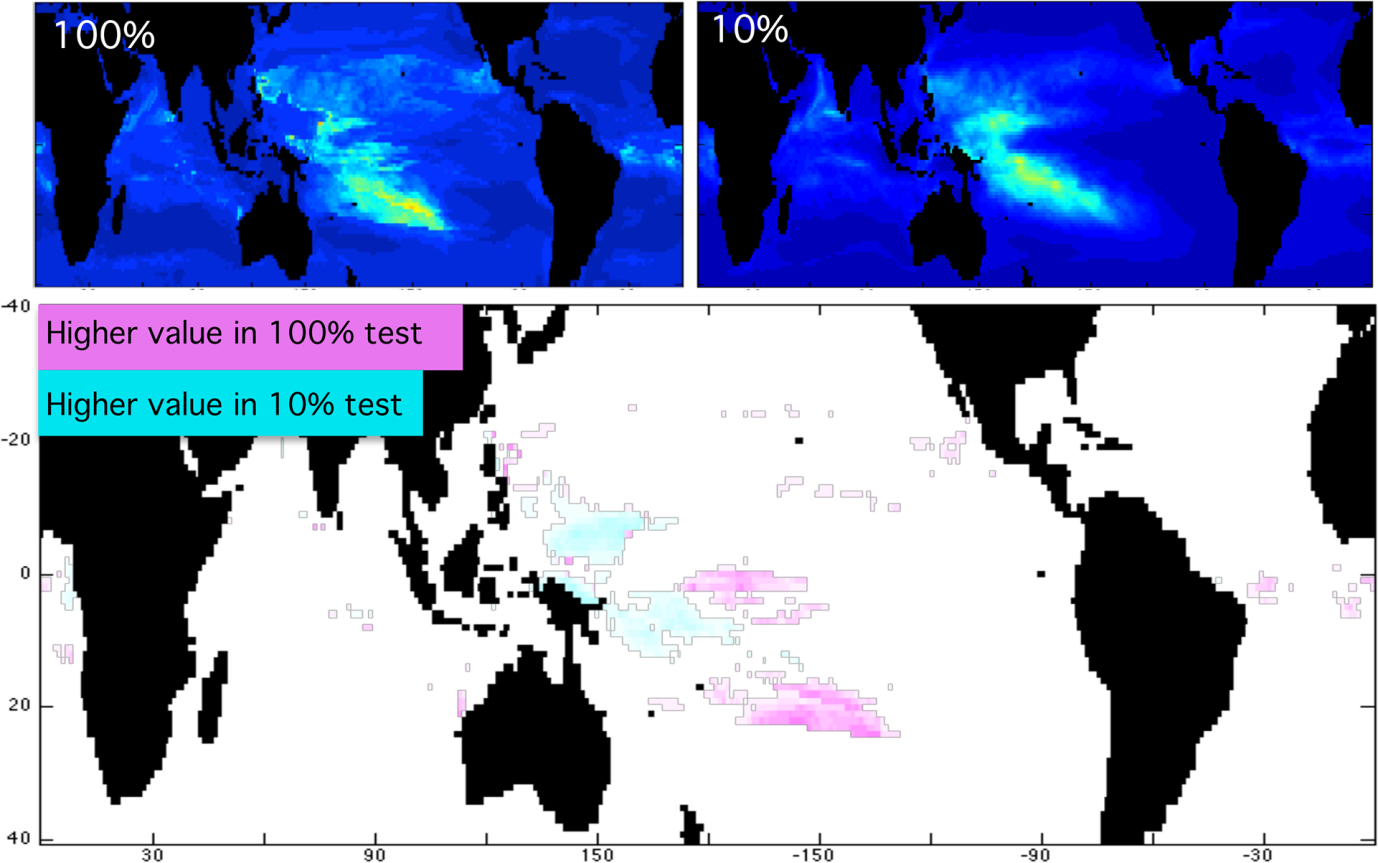
Comparison of Habitable Space From Tests Using All and Only 10% of Remote Pacific Reef Locations. A direct comparison of habitable space projections using 100% (top left) and 10% (top right) of the remote Pacific reef locations for training Maxent and subsequent calculations is shown in the maps above. Color scale is identical to Figs 3–4 and indicates habitat suitability from most (red) to least suitable (dark blue). Note the similarity in over-arching pattern of both maps above. The numerical difference between the maps is shown below. Differences <0.1 are white. Higher values in the 100% test are shown in pink (darker hues for larger values), and higher values in the 10% test are shown in blue (darker hues for larger values).

Tests using 10–90% of all remote pacific reefs were conducted to determine effect of sample size on result. Mean and standard deviation do not vary significantly between these tests for 2030, 2060, or 2090 (Fig 5) indicating that even just 30 points is enough to provide some insight to coral reef habitat and potential habitat throughout the tropical oceans. Far more meaningful than the mean value is the geographic pattern of potential habitable space in future climate scenarios. Fig 6 shows a direct comparison of such patterns obtained using 100% of the remote Pacific reefs for training and 10% of remote Pacific reefs for training. While there are small differences, it is clear that the overarching pattern is the same in both cases. The test case using more (100%) of coral locations for training shows a more complex structure of suitable habitat in future climate scenarios, and picks up on a few small areas that are not identified in the test using few (10%) coral locations for training. The geographic pattern and trends in habitable space across the tropical oceans are what the conclusions in this paper are drawn from. It is preferable to use the largest number of locations for model training to produce the most complete projection of habitable space in future climate scenarios. This study aims to use smaller selections of locations for model training that are more tightly constrained in their physico-chemical parameters to provide insight as to how specific coral reef habitats respond to climate change forcing. Given the results of the sample size testing, it is important to base conclusions on the large-scale trends found here, not the response at one location.

## Discussion

Projections of future coral reef habitat suitability for narrow physico-chemical environments revealed several key trends. Habitats considered marginal today are robust in future projections. Other habitats that are not presently considered marginal rapidly disappear in model projections, which are described as ‘fragile habitats.’ A climate change refuge the South Pacific is predicted to experience moderate shifts in physico-chemical environmental variables, but remain within the broader envelope of coral reef tolerance.

### Robust Persistence of Marginal Coral Reef Habitats

Two habitats that are considered marginal today–the High Latitude reefs and Equatorial reefs (including the Eastern Pacific) (Fig 4) are projected to persist and expand in future climate projections. Both habitats are characterized by relatively cooler water, higher nutrient levels, and lower aragonite saturation state. The suitable habitat associated with High Latitude reefs extends into the South Pacific Refuge, while the Equatorial habitat does not. The High Latitude reef habitat is pre-conditioned to lower aragonite saturation state, which is then a major driver for future projections of habitable space. High temperatures in the western Pacific prevent both of these habitats from extending into the western Pacific coral triangle region (Fig 4). The High Latitude reefs are also pre-conditioned to strong currents, low nutrient levels, and a large seasonal temperature range (Table 2). All of these features likely drive the persistence of habitable space for the High Latitude Reefs in western boundary current systems in future climate scenarios. Furthermore, the High Latitude reefs tests highlight suitable habitat in the coastal Caribbean basin. This is a sharp contrast to previous results considering coral reefs in each ocean (Pacific, Caribbean, Indian), where even training Maxent with Caribbean reefs resulted in little to no suitable habitat in the Caribbean basin [28]. The high salinity, seasonal temperature range, and thermal stress are the main factors behind this emergent suitable habitat in the Caribbean.

These results are promising for the future of coral reefs as a whole. However, it is important to note that especially for the Equatorial reefs, the large range of suitable habitat covers regions that are too deep to be inhabited by coral reefs, which require access to sunlight. The more meaningful result for the Equatorial reefs is the persistence of the same physico-chemical environment for the specific coral reef locations under climate change forcing. Similarly, the High Latitude reefs maintain a strong habitable space in the current coral reef locations, with some expansion equator-ward (especially in the Southern Hemisphere). The most limiting factor for both of these regions will almost certainly be aragonite saturation state [14]. While this was found to not change dramatically from late 20^th^ century values in this study (in NCAR CESM1 projections), these reefs have been previously considered marginal because they are living near their physiological limit of aragonite saturation state value [19].

### Fragile Habitats Are Rapidly Lost in Climate Change Scenarios

Two distinct habitats rapidly vanished from the tropical oceans in climate change scenarios, described here as ‘fragile habitats.’ The Northwest and Central South regions would not be considered marginal by classical definition, however the combination of oceanographic conditions that define these unique habitats will rapidly change in these regions. Notably, both of these regions are characterized by a small annual temperature range (summer–winter) and low thermal stress (DHW). These temperature metrics are one of the first to shift in the NCAR CESM-1 projections, and are the most significant factor for the loss of habitable space for coral reefs in the Northwest and Central South regions.

This result sounds grim, but rather indicates that Central South and Northwest coral reefs will experience a shift in temperature, thermal stress, current speeds, aragonite saturation state, and nutrient levels. The shift in these combined variables will result in a new physico-chemical environment. When the tropical Pacific is considered as a whole, both of these regions have persistent suitable habitat under climate change scenarios [28]. The key finding here is that the habitat will change, while still remaining in the envelope of broad-scale coral reef tolerance.

### Shift in the South Pacific Refuge

The results presented here reinforce the previous finding of a key refuge in the south Pacific around French Polynesia [28], which is repeatedly predicted as a suitable coral reef habitat in future climate scenarios. Further investigation toward what drives the presence of this refuge shows that the current oceanographic state of this region does not remain stable in climate change scenarios. However, the area reaches a new state in global change projections by model year 2010–2020 that remains similar to the conditions experienced by much of the Pacific and Indian Oceans today throughout the 21^st^ century. While this region is an important area to explore for the sake of conservation efforts [42], the ability of coral reefs within the region to ecologically adapt from their current physico-chemical habitat to another (similar to many coral reef physico-chemical habitats nearby) is a critical question that needs to be addressed.

### Advantages and Limitations of Maxent

The random percent tests showed that a large sample is not required for using Maxent to test suitable habitat for coral reefs throughout the tropical sea. This result allowed for the testing of geographically smaller, narrower physico-chemical habitats with Maxent. Testing across narrower ranges of physical and chemical variables should give more robust projections. Even so, some reviews have questioned the validity of using SDMs such as Maxent to accurately predict species presence [43–44]. The critical constraint is that the projections shown here are based solely on changes in physical and chemical variables of the coral reef environment. There is no allowance for corals to adapt. There is no consideration of sea level rise, particularly in conjunction with reduced calcification rates, which may lead to reef loss. The current study does not consider future projections of cyclones or rainfall patterns, aside from nutrient levels including runoff in the atmosphere-ocean coupled earth system model (CESM1). The projections shown here are most useful when considered as a gradient of habitat suitability–not a prediction of species presence [45]. Furthermore, the results presented here are based on the projections of a single earth system model. However, these projections are at least qualitatively useful in demonstrating the nature of the shifts in bioclimatic envelopes for coral reef ecosystems, and provide insights into the persistence or lack thereof for particular physico-chemical coral reef habitats in climate change scenarios.

### Applying Study Results to Conservation

Maxent does not consider the state of coral reefs in an area, only the presence or absence. The results presented here should be used to identify locations where coral reefs may potentially survive in present and future climates, not as an indication of current or future coral reef health. That said, reefs in certain oceanographic habitats that quickly vanish under climate change scenarios will likely experience far more environmental stress than those in more persistent habitats. This consideration is critical when planning large management and conservation efforts, as the climate-driven shifts in sea surface temperature anomalies and pH are far beyond the scope of most governments and conservation organizations to manage. A triage approach would focus on those reefs located in the more persistent oceanographic habitats such as the High Latitude and Equatorial Reef regions. The projections discussed here may also be an important guide for any ‘re-wilding’ or ‘managed relocations’—transplanting healthy corals to persistent refugia to ensure the continuation of a species [46]. Given the dependence of the diverse coral reef ecosystem on the structural complexity provided by reef-building corals, some scientists and conservation organizations have considered introducing non-native species of reef-building corals to likely refugia to maintain ecosystem function. [46–47]. Re-wilding, managed relocations, and species are all controversial topics [48]. Should a major reef-building species decline due to factors associated with climate change; it may be advantageous to introduce an alternate reef-building species that is more suited to the new environment to maintain reef functionality. A recent study showed that trans-located corals could develop high heat tolerance within two years [49].

## Conclusion

This study utilized earth system model projections from the NCAR CESM-1 biogeochemistry runs in conjunction with the species distribution algorithm Maxent to determine which Pacific coral reef regions are pre-conditioned to future oceanic habitats, and which will experience a high degree of change in their oceanic habitat. Sample size testing for Maxent indicated that even small habitats could produce reliable projections. Previous studies have shown a general constriction of suitable habitat throughout the 21^st^ century for all Pacific coral reefs, and an expansion of suitable habitat for all Indian Ocean coral reefs [28]. This study refined those results by considering seven unique habitats individually for the Maxent algorithm, which provided a region-specific view of the effects of climate change on specific oceanic regions. Contrary to expectation, habitats that are currently considered ‘marginal’ (the High Latitude reefs in both hemispheres, and the Equatorial/Eastern Tropical Pacific reefs) have an impressive increase in habitable space in the 21^st^ century (Fig 4). Other regions that would be considered ‘ideal’ for coral reefs today rapidly lost nearly all of their habitable space in climate change projections, as a result of being characterized by a narrow seasonal temperature range and low thermal stress. The remainder of the Pacific Reefs, including the West Pacific Warm Pool/Coral Triangle region showed general constriction of habitat throughout the 21^st^ century, more pronounced for the business-as-usual emissions scenario RCP8.5. The future state of the key South Pacific Refuge around French Polynesia is similar to the physico-chemical environment of higher latitude reefs today.

## Supporting Information

S1 File Freeman et al. 2012 Manuscript. Classification of remote Pacific coral reefs by physical environment by Lauren A. Freeman, Arthur J. Miller, Richard D. Norris, Jennifer E. Smith. This study uses several physico-chemical variables to categorize remote Pacific coral reefs into unique habitats. Those seven habitats are the input data for the current study.
(PDF)

S2 File Freeman et al. 2013 Manuscript. Coral Reef Habitat Response to Climate Change Scenarios by Lauren A. Freeman, Joan A. Kleypas, and Arthur J. Miller. This study sets precedent for the current study in use of a coupled earth system model (CESM1) with the species distribution algorithm Maxent to make future projections of coral reef habitat suitability.
(PDF)
