# Supplementary material for: Robust Performance of Marginal Pacific Coral Reef Habitats in Future Climate Scenarios
Source: PLoS One. 2015 Jun 8;10(6):e0128875. doi: 10.1371/journal.pone.0128875 (PMC4459991; doi:10.1371/journal.pone.0128875)
Supplement: S1 File — Classification of remote Pacific coral reefs by physical environment by Lauren A. Freeman, Arthur J. Miller, Richard D. Norris, Jennifer E. Smith. This study uses several physico-chemical variables to categorize remote Pacific coral reefs into unique habitats. Those seven habitats are the input data for the current study. (PDF) [file pone.0128875.s001.pdf]

JOURNAL OF GEOPHYSICAL RESEARCH, VOL. 117, C02007, doi:10.1029/2011JC007099, 2012

Classification of remote Pacific coral reefs by physical oceanographic environment

Lauren A. Freeman,[1] Arthur J. Miller,[1] Richard D. Norris,[1] and Jennifer E. Smith[1]

Received 6 March 2011; revised 30 November 2011; accepted 1 December 2011; published 2 February 2012.

[1] The oceanographic environment is a key element in structuring coral reef ecosystems by setting the range of physical and chemical conditions in which coral reef-builders live. A cluster analysis of physical and chemical oceanographic data is used to classify coral habitats in the remote tropical and subtropical Pacific Ocean based on average temperature, temperature seasonal cycle, nutrient levels, salinity, aragonite saturation state, storm frequency, intense hurricane hits, and dissolved oxygen as well as temperature anomalies in degree heating weeks. The resulting seven geographic habitats are stable to perturbations in types of data used in the cluster analysis. Based on recent coral reef survey data in the area, the coral cover was related to the identified geographic regions. The habitats tend to be geographically clustered, and each is characterized by a unique combination of oceanographic conditions. Previous studies suggest coral reef habitats are associated with a uniform array of oceanographic conditions, while our results demonstrate that finer-scale variations in physical variables may control coral reef environments. The results better define the physical environment of remote coral reefs, forming a foundation for future work addressing physical habitat perturbation and anthropogenic impacts on reefs.

Citation: Freeman, L. A., A. J. Miller, R. D. Norris, and J. E. Smith (2012), Classification of remote Pacific coral reefs by physical oceanographic environment, *J. Geophys. Res.*, *117*, C02007, doi:10.1029/2011JC007099.

1. Introduction

[2] A classical paradigm for coral reefs is that they grow in a fairly uniform oceanic environment: warm, oligotrophic, and low kinetic energy [*Stoddart*, 1969]. However, decades of research in both coral reef ecology and tropical oceanography have suggested that there is truly a large dynamic range of physical oceanographic conditions in which they can thrive [*Ando and McPhaden*, 1997; *Glynn and Colgan*, 1992; *Glynn and Ault*, 2000; *Johannes et al.*, 1983; *Rosen*, 1988; *Wyrtki*, 1975; *Zhang*, 1996, 2005].

[3] A cursory view of the physical and chemical oceanographic conditions around where reef-building corals grow indicates that there exists a broad range of ambient nutrient levels, sea surface temperature (SST), annual variability, storm intensity and frequency, and other factors. For instance, hermatypic coral reefs are known from a broad array of temperatures (17–29°C), and nitrate concentrations (0–13 mmol/m^3). Anomalies in the physical environment can result in stress on the ecosystem. This is best documented with temperature. In fact, changes in the physical environment are included in ecological definitions of disturbance (i.e., disturbance can be defined as “any relatively discrete event in time that disrupts the ecosystem, community, or population structure and changes resources, substrate availability, or the physical environment” [*Pickett and White*, 1985]). To assess such anomalies, one must first understand the average climatology for a region.

[4] Temperature is a fundamental control on coral reef ecosystems. Temperature variability, in particularly prolonged abnormal warm temperatures, result in coral bleaching [*Glynn*, 1996], which often leads to mortality and subsequent reduction in coral cover. Coral reef locations are strongly controlled by temperature, and reefs do not grow where mean SST drops below 17°C. There are likely large differences in ecosystem structure between reefs with low and high temperature variability, as with terrestrial biomes. For this reason, our analysis includes annual mean SST (which sets the upper latitudinal limits of coral reefs), annual seasonal cycle, and daily anomalies in the form of degree heating weeks (DHWs). The mean and seasonal cycle information relates directly to the “mean” ecosystem state, whereas DHW relates to stress events and disturbance.

[5] Aragonite saturation state (Ω-arag) tends to be higher in warmer water and lower at higher latitudes. It is a key determinant of the upper reach of coral reef development. Corals produce aragonite skeletons, and Ω-arag is directly related to rates of calcification. In current conditions, most reefs grow slowly (∼1 cm per year), and decreases in Ω-arag from increased CO_2 emissions are a serious concern for future coral reef development [*Hoegh-Guldberg*, 2011; *Hoegh-Guldberg et al.*, 2007].

[6] Nutrients (phosphate, nitrate, and silicate) and oxygen are limiting factors for photosynthesis (nutrients only) and

[1]Scripps Institution of Oceanography, University of California, San Diego, La Jolla, California, USA.

Copyright 2012 by the American Geophysical Union.
0148-0227/12/2011JC007099

respiration (both nutrients and oxygen), and the relative concentrations of these resources will partially dictate whether ecosystems are dominated by autotrophs or heterotrophs. We hypothesize that both nutrient levels and oxygen levels are fundamental in structuring the physical environment for coral reef ecosystems. Nutrients are of particular interest as corals are adapted to thrive in low-nutrient environments [*Muscatine and Porter*, 1977], and may suffer a competitive disadvantage to faster growing algae in the case of increased local nutrient availability [*Szmant*, 2002].

[7] Cyclones have been documented as intermittent, severe stress events on coral reefs. While storms are relatively rare, and catastrophic storms are extremely rare, the damage that they cause is long lasting. Major effects on coral reefs include debris from land, increased sedimentation from land that may smother corals, and physical breaking and repositioning of coral reef superstructure [*Bythell et al.*, 1993, 2000; *Dollar and Tribble*, 1993; *Edmunds and Witman*, 1991; *Gardner et al.*, 2005; *Harmelin-Vivien and Laboute*, 1986; *Woodley et al.*, 1981]. We theorize that storm frequency is related to reef morphology, where massive boulder corals likely dominate reefs with high incidence of storms and reefs with rare storms are likely dominated by faster growing but more delicate branching corals. In addition to morphology, storm events may be related to biodiversity and ecosystem function via the intermediate disturbance hypothesis [*Connell*, 1978; *Rogers*, 1993] wherein the highest diversity of reef corals would be expected in areas with intermediate levels of storm frequency and/or intensity. Furthermore, there is evidence that cyclone intensity and damage will increase with continued anthropogenic climate change [*Emanuel*, 2005].

[8] The environmental context of coral reefs must be recognized in order to objectively compare and contrast geographically disparate sites. We attempt to address this issue here by objectively identifying coral reef habitats based on physical and chemical oceanographic data. We use a cluster analysis that includes SST, DHW, Ω-arag, nutrient, oxygen, and storm metrics. We restrict our analysis to the Pacific Ocean because of the remoteness of coral reefs in this basin. This analysis for current climate conditions can then serve as a baseline for estimating coral reef survival capabilities in studies of future climate scenarios.

[9] In previous research, *Kleypas et al.* [1999] attempted to identify the range of conditions in which hermatypic reefs can survive in order to identify "marginal reefs" which are near the edge of their reach and may be threatened by changing environmental conditions. They used a cluster analysis to identify such reefs, which they determined were best described by extremes of high temperature, low light availability, and low Ω-arag. They also identified "no-worries reefs," which were situated in less severe environmental conditions. In the Pacific Ocean, the "no-worries" reefs covered essentially the entire remote open-ocean area, which is the subject of the current study.

[10] Here we explore the oceanographic diversity of the *Kleypas et al.* [1999] "no-worries reefs" in the Pacific by considering variations within the broad window of environmental variables in which the coral reefs grow. We expand the list of variables previously used by *Kleypas et al.* [1999], and instead of identifying marginal reefs at risk of decline from a changing environment, we seek to understand the current oceanographic state of reefs in the tropical Pacific and the variability of oceanographic conditions between reefs.

[11] The only variable omitted from our study that was included in the Kleypas study was light availability. *Kleypas et al.* [1999] found that low light availability was indicative of marginal reef environments. In the Pacific, these marginal reefs were almost entirely coastal and likely related to terrestrial sedimentation influx. We have already excluded the reefs characterized by *Kleypas et al* [1999] as light-limited from our study by removing coastal reefs. The only two exceptions occurred at high latitude in the south Pacific, but these reefs are strongly differentiated from other locations by low temperature, strong seasonal cycle, and low Ω-arag in the current study.

[12] It is well known that coral reefs are declining because of many factors [*Hoegh-Guldberg*, 1999] including climate change and associated variability. These climate-induced impacts have been documented extensively both in laboratory experiments and in the field [*Hoegh-Guldberg*, 2009]. It is also known that coral reefs have some ability to adapt to climate change. For example, corals adapt better to gradual environmental changes than extreme events, as seen in shifts toward more heat-tolerant zooxanthellae and recovery following bleaching episodes [*Baker et al.*, 2008, 2004]. However, the resilience of reef communities may also be linked to synergistic effects of an individual reef's environment: long-term temperature changes, short-term temperature anomalies, varying nutrient levels, and other fluctuating physical oceanographic variables.

[13] We suggest that the first-order structure of reef communities (e.g., abundance of reef-building corals) is likely set by the physical environment: both the mean conditions of temperature, nutrients, and salinity as well as the range of environmental variability and propensity toward extreme conditions in different oceanic environments. We start with this assumption to conduct a quantitative classification of the physical environment around Pacific coral reefs and then examine coral cover data to test our assumption.

2. Materials and Methods

[14] The physical and chemical variables of interest are outlined in Table 1 along with all open-ocean coral sites from ReefBase in the Pacific. A cluster analysis of the physical variables at the coral sites is then performed to identify similar reef environments. Coral cover is then independently compared to the habitats identified by the clusters.

[15] Physical and chemical oceanographic data from the World Ocean Atlas (WOA) and NOAA were obtained on a uniform global grid of 1° latitude $\times$ 1° longitude (180 $\times$ 360, 110 $\times$ 110 km) resolution. This grid was selected to match the finest-resolution climatology product available from WOA. Sea surface data including temperature, phosphate, dissolved oxygen, and salinity were incorporated [*Antonov et al.*, 2006; *Garcia et al.*, 2006a, 2006b; *Locarnini et al.*, 2006]. The seasonal range of temperature was calculated as absolute value of summer-winter difference from WOA seasonal climatology. Phosphate is used as a proxy for all nutrients, as it is the most completely measured nutrient in the WOA and is correlated with nitrate [*Cooper*, 1937]. We

Table 1. Description of Physical and Chemical Oceanographic Variables Used in the Cluster Analysis

Variable	Data Source	Notes	Type
Average sea surface temperature	World Ocean Atlas [*Locarnini et al.*, 2006]	Cumulative average from early 1900s	Cruise data
Average sea surface phosphorus	World Ocean Atlas [*Garcia et al.*, 2006a]	Cumulative average from early 1900s	Cruise data
Average sea surface dissolved oxygen	World Ocean Atlas [*Garcia et al.*, 2006b]	Cumulative average from early 1900s	Cruise data
Average sea surface salinity	World Ocean Atlas [*Antonov et al.*, 2006]	Cumulative average from early 1900s	Cruise data
Average annual temperature variability	World Ocean Atlas [*Locarnini et al.*, 2006]	Difference between warmest average month and coldest average month	Cruise data
Temperature anomaly - Degree Heating Weeks	NOAA Coral Reef Program	Average DHWs from 1985–2010	Satellite data
Average aragonite saturation state	GLODAP [*Key et al.*, 2004], WOA [*Antonov et al.*, 2006; *Locarnini et al.*, 2006]	Calculated aragonite saturation state from 1985–2010	Satellite + cruise data
Annual storm frequency	NOAA Historic Hurricane Tracks	Includes all cyclonic storms within 110 km diameter cell	Satellite data + observations
Intense hurricane hits	NOAA Historic Hurricane Tracks	Category 4 and 5 hurricanes within 110 km diameter cell	Satellite data + observations

considered nutrients from WOA to be more representative of upwelling patterns than a data set obtained by calculating regional upwelling from Ekman stress.

[16] We considered three temperature metrics: mean temperature, annual temperature variability, and DHWs, as temperature and temperature variability have been repeatedly associated with coral reef environments and stress events. DHWs time series data for each point with coral reefs were collected from 1985 to 2010 and averaged. DHWs are the cumulative sum of temperature anomalies greater than 1°C above the warmest temperature in the mean monthly climatology. NOAA DHWs were used, which are calculated weekly twice from advanced very high-resolution radiometers on board NOAA's Polar-Orbiting Operational Environmental Satellites. Then grid points were aligned with the WOA data, corresponding to an approximate grid point resolution of 110 × 110 km.

[17] The value of Ω-arag was calculated from Global Data Analysis Project, dissolved inorganic carbon and alkalinity [*Key et al.*, 2004], and WOA temperature and salinity [*Antonov et al.*, 2006; *Locarnini et al.*, 2006] using CO2SYS in MATLAB for the surface ocean at 1° latitude × 1° longitude resolution.

[18] Cyclonic storm data were collected from the geographic information system (GIS)-based NOAA historic hurricane track tool (http://www.csc.noaa.gov/hurricanes/). This stores information on cyclonic storms from 1982 to present, including tracks centered at the storm's eye, storm category, and storm name from a combination of satellite data and observations. Each location was queried with a 55 km radius (to match the 110 km grid cells of 1° latitude × 1° longitude), for all months from January 1985 to December 2010. The 55 km radius is also consistent with the "buffer" from storm centers used in a previous meta-analysis [*Gardner et al.*, 2005]. These dates were selected to align with the DHWs data. For each location, the total number of cyclones that passed through the 110 km diameter cell over 25 years was recorded to calculate annual storm frequency, as well as the number of hurricanes that reached category 4 or 5 on the Saffir-Simpson wind scale (sustained winds of 131–155 mph, and greater than 155 mph, respectively) while inside of that particular cell (intense hurricane hits). NOAA describes both these categories as "catastrophic damage will occur" categories. For reference, a category 3 hurricane has sustained winds of 111–130 mph and is described as "devastating damage will occur."

[19] These data are representative of local oceanographic conditions in diverse coral reef regions of the Pacific. We recognize that smaller-scale physical oceanographic variations are important for coral reef habitats, which have very fine-scale structures, even within a single reef [*Selig et al.*, 2010]. But our philosophy here is to focus on the regional scale structures that can serve to classify and categorize broad-brush groupings of coral habitats for which this resolution is appropriate.

[20] Each data set was normalized to be unitless and have a mean of 0 and standard deviation of 1 so that all variables were weighted equally in analyses [*Lewis et al.*, 2008]. Coral reef locations were determined from ReefBase GIS maps (http://www.reefbase.org/) and extrapolated to a 1° latitude × 1° longitude grid for a total of 305 cells. Barrier reefs or reefs near landmasses were not included in this analysis because of a paucity of oceanographic data available at these points as well as poorly resolved satellite land/sea correction resulting in less reliable satellite data. High-latitude rocky reefs with solitary corals were also excluded. For each cell (1° × 1°), each physical and chemical variable was extracted and stored.

[21] A cluster analysis was performed on these coral reef cells using *k*-means clustering. The *k*-means clustering is an iterative loop that determines clusters by cumulative root mean square distance between points, for a set number of clusters (k). The maximum point to centroid (mean value of all variables for the cluster) distance was calculated for k = 1–20 total clusters. The point-to-centroid distance is a measure of strength of the cluster analysis (a smaller point-to-centroid distance indicates more tightly confined clusters or habitats). To minimize variance within habitats defined by our nine data metrics we used the k = 7, which had minimum point-to-centroid distance, for final calculations.

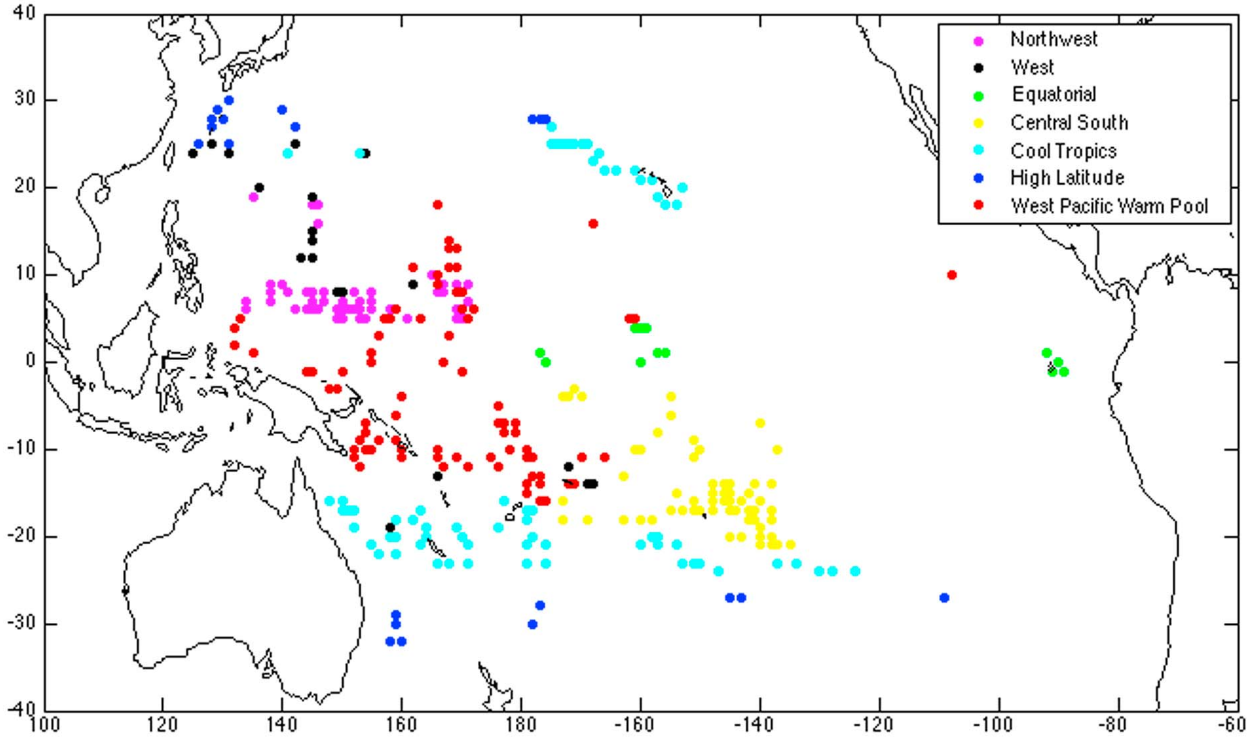

Figure 1. The seven coral habitats mapped spatially with each habitat as a different color.

All calculations were performed in MATLAB Version 7.12.0, 2011 release.

[22] To compare an independent measure of coral reef health with the cluster analysis habitat results, we chose percent coral cover as the comparative metric because of its wide availability and common use in previous studies to indicate the status of a given coral reef. Percent coral cover is also often associated with a number of biological variables such as macroalgal cover and herbivore abundance [*Graham et al.*, 2008; *Hoegh-Guldberg*, 1999; *Sandin et al.*, 2008]. These ecological data were collected from the World Atlas of Coral Reefs [*Spalding et al.*, 2001], NOAA Coral Reef Ecosystem Division [*Waddell and Clark*, 2008], the Global Coral Reef Monitoring Network [*Salvat*, 2002; *Wilkinson*, 2008], and from recent observations obtained in field surveys. If multiple data points were available within a 1° latitude × 1° longitude cell, they were averaged. These particular data are island- or reef-wide averages obtained via multiple surveys and/or photo transects. To minimize discrepancies between collection methods, coral cover percentages were discretely binned into 10 categories. While this ecological data set does not cover the entire set of coral reef locations used in the cluster analysis of physical habitats, it is extensive enough to provide a cursory insight into the relationship between coral habitats and percent coral cover. We recognize the potentially serious deficiencies in this data set, including long-term trends because of non-physical oceanographic stressors (e.g., local human population changes, fishing, and pollution) and small-scale variations in percent coral cover [*Edmunds and Bruno*, 1996]. This is not intended to act as a meta-analysis of percent coral cover data but rather to serve as an initial ecological comparison with our analysis of physical oceanographic data.

3. Results

[23] The cluster analysis produced seven habitats based on nine variables (Figure 1). The average normalized values for each habitat are described in Table 2. The clusters were not uniform in size. The number of locations per cluster and number of locations with coral cover data per cluster are shown in Figure 2. Each habitat was characterized by a different combination of the nine oceanographic variables, as described here. All the locations considered except two were classified as “no-worries” by *Kleypas et al.* [1999]. By considering several additional environmental variables, we have been able to show distinct geographic groupings of coral reef locations based on their physical environment. While latitude gives a rough approximation of oceanographic conditions, our results show that there is also a large degree of zonal variation.

[24] Hurricane distribution was nonrandom with respect to both latitude and longitude. Annual storm frequency was 2–3 times greater in the Northern Hemisphere than the Southern, and showed a linear decline from the West to the East (Figure 3). The difference between hemispheres is also reflected in a previous study of hurricane intensity [*Emanuel*, 2005], and increase in intensity from the East to the West is expected from SST patterns. One point in the eastern Pacific also experienced high storm frequency as a result of cyclones that were formed in the Caribbean traveling across Central America. No hurricanes occurred within 3° of the equator because of weak Coriolis force at low latitudes.

Table 2. Description of Each Habitat by Centroid

Habitat	Annual Storm Frequency	Intense Hurricane Hits	Salinity	Phosphate	Seasonal Temperature Range (°C)	Oxygen	Ω-arag	DHW (°C)	Mean Temperature (°C)
Exact Values in Original Units									
Northwest Pacific	0.62	0.00	34.23	0.13	0.91	4.54	3.95	0.19	28.90
West Pacific	0.61	1.11	34.67	0.12	2.84	4.60	3.91	0.31	27.74
Equatorial Pacific	0.00	0.00	34.86	0.55	1.84	4.63	3.59	1.62	26.31
Central South Pacific	0.04	0.00	35.84	0.29	1.78	4.63	4.15	0.17	27.63
Cool Tropics	0.14	0.00	35.29	0.14	3.51	4.76	3.85	0.27	25.50
High Latitude Reefs	0.28	0.00	35.14	0.12	5.92	4.93	3.60	0.46	23.37
West Pacific Warm Pool	0.13	0.00	34.60	0.20	1.10	4.55	3.87	0.22	28.70
Descriptive Values From Cluster Analysis Result[a]									
Northwest Pacific	High	Average	Low	Low	Low	Low	Average	Average	High
West Pacific	High	Very High	Low	Low	Average	Average	Average	Average	Average
Equatorial Pacific	Low	Average	Average	Very High	Average	Average	Low	Very High	Low
Central South Pacific	Low	Average	High	High	Average	Average	High	Average	Average
Cool Tropics	Average	Average	Average	Average	High	High	Average	Average	Low
High Latitude Reefs	Average	Average	Average	Low	Very High	Very High	Low	High	Very Low
West Pacific Warm Pool	Average	Average	Low	Average	Low	Low	Average	Average	Average

[a]Descriptive values were defined from normalized variables (zero mean, unit standard deviation) as “very low” >−2, “low” = −2 to −0.5, “average” = −0.5 to 0.5, “high” 0.5 to 2, “very high” >2. These give an indication of conditions in each cluster relative to all of the coral reef locations used in the analysis.

3.1. Northwest Pacific

[25] The northwest Pacific habitat is completely located between 5°N–20°N and 130°E–180°E (Figure 1) and includes most of the Federated States of Micronesia, notably Palau. It is characterized by the highest annual storm frequency and mean temperature of all habitats and a low seasonal cycle and nutrients. The storms and mean temperature differentiate this habitat from the west Pacific warm pool, which experiences slightly lower values of each. The mean temperature in the northwest Pacific is 28.9 °C, and the seasonal cycle is less than 1°.

3.2. West Pacific

[26] The west Pacific habitat is the most scattered geographically, from 120°E to 160°W and from 20°S to 25°N (Figure 1), and includes Guam and the Northern Mariana Islands. It is characterized by intense hurricane hits and high annual storm frequency. The only locations with intense hurricane hits in the past 25 years fall into this cluster. In addition to receiving intense storms, these locations have the second highest annual storm frequency (0.61 storms per year versus 0.62 in the northwest Pacific), so they receive both regular and severe disturbance.

3.3. Equatorial Pacific

[27] The equatorial Pacific habitat has the smallest number of points (12), and includes the Galapagos Islands and other central Pacific reefs, all within a few degrees of the equator. This habitat has the highest values of phosphorus by far and relatively low temperatures, which can be attributed to regular upwelling along the equator and eastern boundary. These reefs have experienced no hurricanes/cyclones in the past 25 years. Annual temperature variability is also average, although interannual temperature variability would likely be high because of El Niño Southern Oscillation cycles. [*Wyrtki*, 1975]. This habitat has the lowest value of Ω-arag as well as the highest value of DHWs, which may be a result of a weak seasonal cycle overlaid by strong

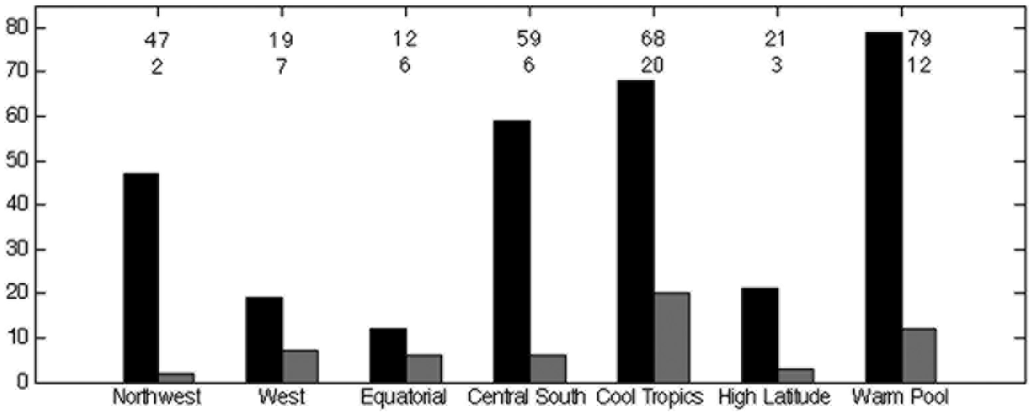

Figure 2. Number of points that contain coral reefs within each habitat (black bars) and number of points that contain percent coral cover data (gray bars). Labels are number of points per habitat (top) and number of points with percent coral cover data per habitat (bottom).

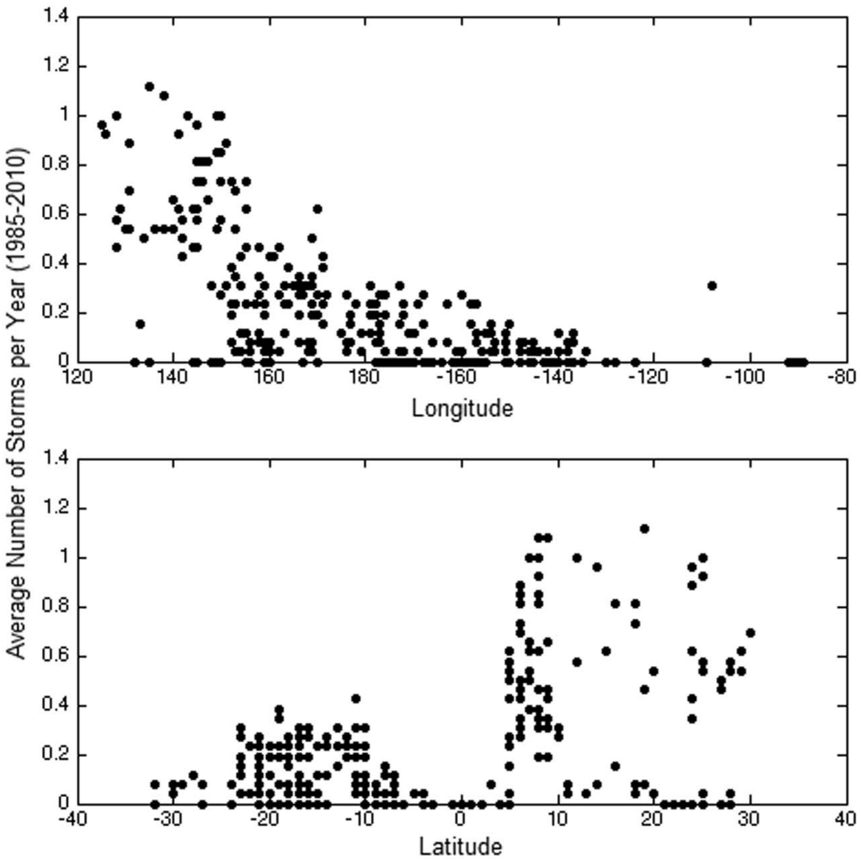

Figure 3. Distribution of cyclonic storms by (top) longitude and (bottom) latitude. Average annual storm frequency was calculated for each data point from 1985 to 2010.

interannual variability. El Niño (or warm) years would result in much higher DHWs values in this region.

3.4. Central South Pacific

[28] The central south Pacific is located exclusively in the Southern Hemisphere, between 130°W and 180°W, and it includes French Polynesia and the Society Islands. It is characterized by high salinity, nutrients, and Ω-arag; low storm frequency; and otherwise average results. It is one of the larger habitats with 59 locations, and it is tightly clustered geographically.

3.5. Cool Tropics

[29] The cool tropics habitat has a strong seasonal cycle and high nutrient levels, low temperatures, and otherwise average results. This habitat includes reefs in the Cook Islands and New Caledonia in the Southern Hemisphere and the main Hawaiian Islands in the Northern Hemisphere. It is differentiated from the central south Pacific by lower Ω-arag, temperature, and nutrient values.

3.6. High-Latitude Reefs

[30] This habitat encompasses the highest-latitude region of all seven habitats, including reefs in the central and western subtropical Pacific. As a result, it is associated with the lowest temperatures and a strongest seasonal cycle of all the habitats. This habitat is further characterized by high oxygen and DHWs values. The higher DHWs values may be a result of lower temperatures in the baseline climatology used to calculate DHWs. It includes part of the northwest Hawaiian Islands, reefs near southern Japan, and remote reefs in the south Pacific such as Lord Howe Island and Easter Island.

3.7. West Pacific Warm Pool

[31] The Warm Pool Tropics habitat is the largest in number of points (79), and it is characterized by a low seasonal cycle. It has low dissolved oxygen and salinity and a midrange value of DHWs. It occurs between 20°S and 20°N, with most of the points grouped to the west. This habitat includes reefs of Papau New Guinea, the Solomon Islands.

3.8. Percent Coral Cover Comparison

[32] Geographic distribution of percent coral cover is shown in Figure 4. A comparison of average coral cover between habitats shows different values for each habitat, although the sample size is very small and standard deviation large (Figure 5). The northwest, central south, and west Pacific warm pool habitats have the highest average coral cover, and the high-latitude reefs have the lowest average coral cover.

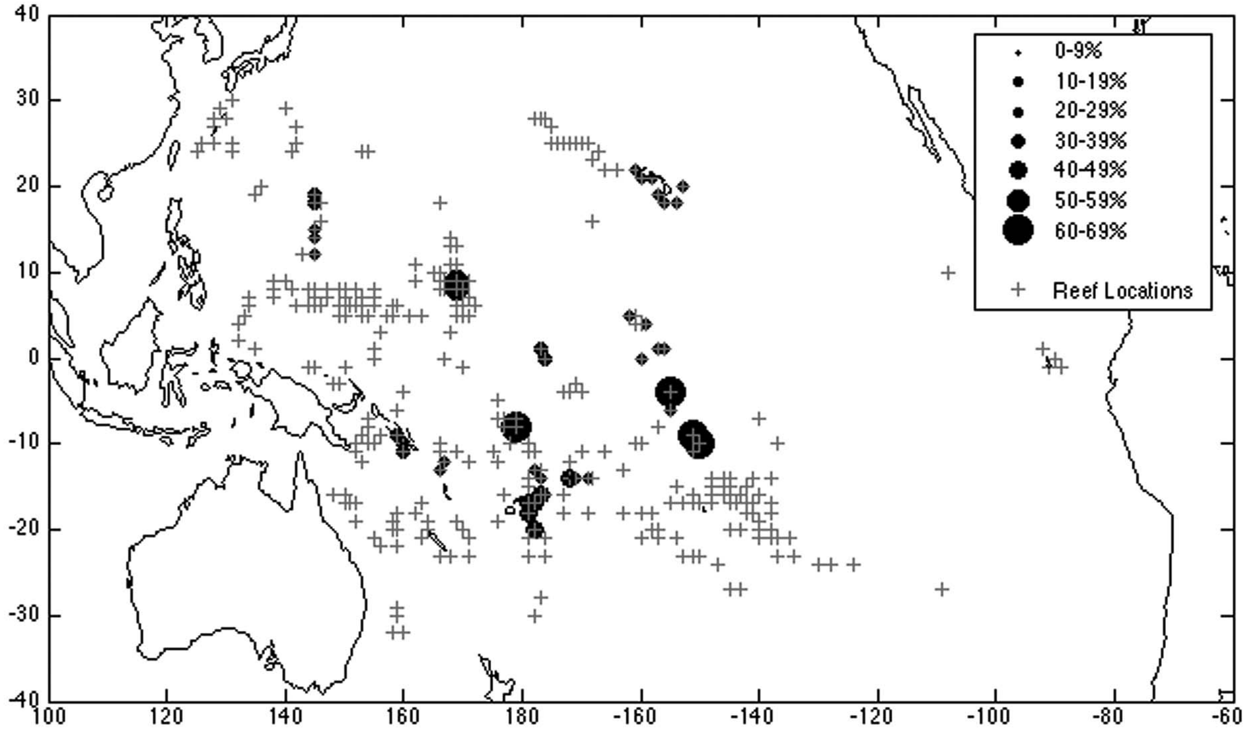

Figure 4. Spatial locations of percent coral cover data from low (small circles) to high (large circles). Each size marker represents a coral cover bin. Coral reef locations considered in this study are marked with gray crosses.

3.9. Sensitivity Analysis

[33] We tested the sensitivity of the cluster analysis in two ways. First, one of the physical variables or variable groups (i.e., all temperature metrics) was removed from the analysis and the cluster analysis was executed with six to nine variables. This process was repeated for each of the six primary variable groups. The clusters in each case (not shown) were very similar to the clusters for the nine variables analyzed together. In comparing the individual results, the designation of locations to specific clusters was between 74% (storms excluded) and 93% (Ω-arag excluded) similar. Second, the cluster analysis was repeated using all nine variables at all open-ocean grid boxes in the tropical Pacific between 35°N and 35°S. Again, the structure of the top

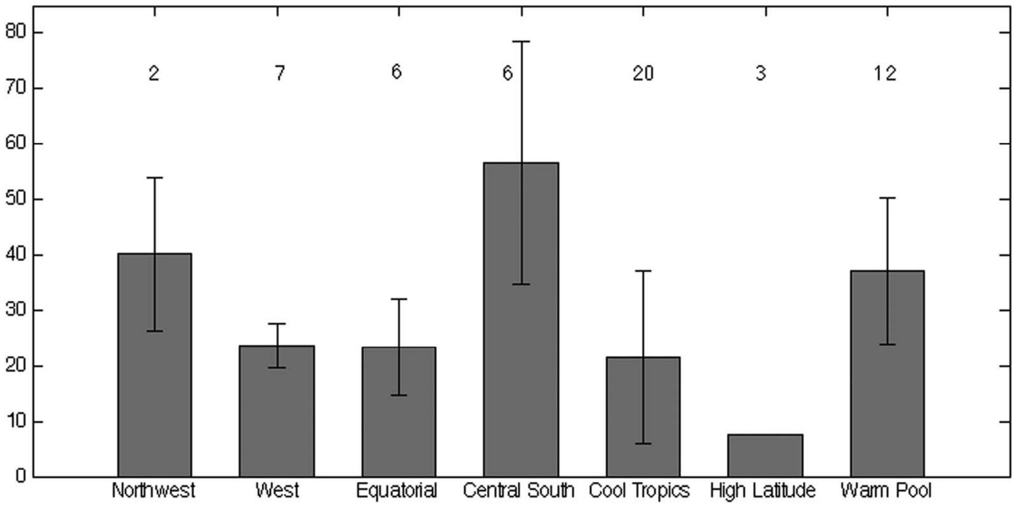

Figure 5. Average percent coral cover within each habitat (gray bars) with 2*standard deviation plotted as error bars. Labels are number of points with percent coral cover data per habitat.

seven clusters was very similar to the results when including only the grid boxes where coral reefs are present indicating the robustness of our analysis.

[34] Variance within habitat (or maximum deviation from habitat centroid) was not related to habitat stability when defining metrics are weighted or removed. The northwest Pacific has the lowest variance within habitat, but also experienced a high rate of exchange with the west Pacific warm pool when variables were shuffled for stability analyses. The west Pacific and equatorial Pacific are by far the most stable habitats, but they have average variance within habitat. The cool tropics and west Pacific warm pool have the highest variance within habitat.

4. Discussion

[35] A cluster analysis of remote coral reef habitats in the Pacific Ocean based on physical and chemical oceanography reveals habitat differences expressed by latitudinal zonation as well as a coarse longitudinal zonation. The reef habitats tend to become differentiated from each other with distance north or south of the equator, as is the case for the distribution of ocean biomes. There is also east-west differentiation into three main groups: eastern, central, and western tropical Pacific habitats. This zonation suggests that the habitats are not just a function of latitudinal temperature gradients. Previous studies have found a strong latitudinal control on oceanic biomes or habitats [*Lewis et al.*, 2008; *Sarmiento et al.*, 2004]. Latitudinal control here seems to be strongly correlated to oceanographic variables that vary across the Pacific basin, in particular temperature variability on an annual and interannual timescales.

[36] Our habitats also show strong similarities to known trends in biodiversity. First-order approximations of biodiversity increase with decreasing latitude and also increase toward the western tropical Pacific [*Bellwood and Hughes*, 2001; *Gaston*, 2000; *Veron et al.*, 2009]. These trends are particularly pronounced for coral reefs [*Willig et al.*, 2003]. Although biodiversity was not included in our analyses, it is interesting to note that other studies have found patterns that appear to be related to our habitats, with the west Pacific warm pool and northwest Pacific having the highest biodiversity and high-latitude reefs having the lowest biodiversity.

[37] The seven oceanographic habitats that were identified here were associated with various levels of coral cover. In particular, the higher-latitude habitats, namely the cool tropics and high-latitude, tend to show low levels of coral cover; and the central south, northwest, and west Pacific warm pool regions show high levels of coral cover. Tropical sites, in contrast, have some of the highest coral cover, although there is considerable variation within these habitats. The northwest Pacific has the highest annual storm frequency and the second highest mean percent coral cover, but the west Pacific has much lower coral cover and all of the intense hurricane hits. DHWs are highest in the equatorial Pacific, followed by high-latitude reefs, both of which have relatively low coral cover. These observations provide some evidence for disturbance and stress in the physical environment affecting coral reef ecosystems. We postulate that the physical environment likely sets the ecological bounds for coral reef development. However, because of the large number of anthropogenic impacts occurring on local scales on reefs today it is difficult to know how much these natural and anthropogenic factors contribute to present-day coral cover on reefs around the world.

[38] The northwest Pacific and west Pacific warm pool habitats displayed a relatively large amount of exchange of points during sensitivity tests. In the final result, much higher storm frequencies and mean SST as well as lower nutrient levels in the northwest Pacific distinguish it from the west Pacific warm pool. The west Pacific habitat was extremely robust and was reproduced exactly in every combination of variables tested except when storm data were excluded. This habitat is dominated by the "intense hurricane hits" metric, and all the reefs that experienced a category 4 or 5 hurricane in the past 25 years are included in this habitat. The equatorial Pacific habitat was also extremely robust, and was reproduced exactly with almost every combination of variables tested. In two tests, it lost three points from its western edge to the west Pacific warm Pool. The central south Pacific showed some fluctuation under stability tests, exchanging points with the cool tropics (from its southern edge) and west Pacific warm pool (from its northwestern corner). Similarly, the cool tropics experienced some exchange with the central south Pacific as well as high-latitude Reefs.

[39] By exploring different combinations of variables, we gained further insight into what was controlling the habitat distribution. Most importantly, the geographic pattern is reproducible using a wide range of oceanographic variables, and it is stable to perturbations in weighting of variables. The habitats characterized by extremes (very high intense hurricane hits in the west Pacific, very high nutrients and DHWs in the equatorial Pacific, very low temperatures in high-latitude Reefs) were the most robust to stability testing. The inclusion of nine physical and chemical oceanographic metrics allows us to better resolve the "buffer zones" at the edges of habitats (i.e., between the northwest Pacific and west Pacific warm pool) by considering as many factors as possible that could affect coral reef ecosystems.

[40] Our results provide a first step in accounting for broad patterns in physical oceanographic parameters around the Pacific in areas where coral reefs exist. The geographic pattern of coral habitats found here provides an interesting setting for oceanographic studies within and between habitats using downscaling from models or finer resolution data products. In addition, these results alongside downscaled oceanographic studies can potentially be used in combination with local anthropogenic factors to examine how these different factors may affect the structure and development of coral reef communities. Our results will thereby strengthen ecological comparisons of coral reefs that range across large geographic areas, in that some of the variance in oceanography can be removed. For example, we found that the large, encompassing category of "no-worries reefs" in the Pacific from *Kleypas et al.* [1999] actually includes a wide range of temperatures, ambient nutrient levels, and variability on annual and weekly scales.

[41] This study of current climate conditions can also serve as a baseline for understanding coral reef survival capabilities in studies where climate conditions change under global warming scenarios. While coral reefs have already changed dramatically from human impacts, understanding their state and oceanographic habitat now allows us to explore the

capacity for coral reefs to survive in future climate scenarios. Future work on this topic must determine what combinations of conditions will be conducive to coral reef survival in the future. For example, what physical environments are appropriate? Where is variability tolerable, and where is it not? How do direct anthropogenic stressors affect coral reef ecology in the different habitats? Our results begin to narrow the range of answers to these questions. Instead of saying "all coral reefs" we can talk about warmer water reefs, cooler water reefs, places where there is a high degree of variability in temperature, and places where the environment is so stable that small perturbations may cause huge responses in the species present.

[42] This study has discretized remote Pacific coral reefs into seven geographic habitats, each of which is characterized by a unique set of physical and chemical oceanographic conditions. These sets of oceanographic conditions define the physical environment, which sets the foundation for understanding ecological disturbance in the form of alteration to or perturbation of the physical environment.

[43] **Acknowledgments.** This work forms a part of the PhD dissertation of L.A.F., who was supported by an NSF IGERT fellowship under the "Global Change, Marine Ecosystems and Society" program at SIO (0903551). L.A.F. received partial support from the joint MCR-SBC-CCE LTER collaboration. A.J.M. gratefully acknowledges funding from NSF (OCE06–47815 and CCE-LTER: OCE-0417616 and OCE1026607) and NOAA (ECPC NA17RJ1231). The views expressed herein are those of the authors and do not necessarily reflect the views of these agencies. The NOAA Coral Reef Ecosystem Division, Hawaii, provided much of the coral cover data. Brian Zgliczynski (NOAA) provided additional coral cover data. Scott Heron (NOAA) assisted with DHWs–time series data. L.A.F. thanks Jeremy Jackson, Stuart Sandin, Joanie Kleypas, and Simon Freeman for helpful discussions during the course of this research. We thank the three referees for important comments that significantly improved the clarity of results.

References

Ando, K., and M. J. McPhaden (1997), Variability of surface layer hydrography in the tropical Pacific Ocean, *J. Geophys. Res.*, *102*(C10), 23,063–23,078, doi:10.1029/97JC01443.

Antonov, J. I., R. A. Locarnini, T. P. Boyer, A. V. Mishonov, and H. E. Garcia (2006), *Salinity, World Ocean Atlas 2005*, vol. 2, 182 pp., NOAA, U.S. Gov. Print. Off., Washington, D. C.

Baker, A. C., C. J. Starger, T. R. McClanahan, and P. W. Glynn (2004), Corals' adaptive response to climate change, *Nature*, *430*(7001), 741, doi:10.1038/430741a.

Baker, A. C., P. W. Glynn, and B. Riegl (2008), Climate change and coral reef bleaching: An ecological assessment of long-term impacts, recovery trends and future outlook, *Estuarine Coastal Shelf Sci.*, *80*(4), 435–471, doi:10.1016/j.ecss.2008.09.003.

Bellwood, D. R., and T. P. Hughes (2001), Regional-scale assembly rules and biodiversity of coral reefs, *Science*, *292*(5521), 1532–1535, doi:10.1126/science.1058635.

Bythell, J. C., M. Bythell, and E. H. Gladfelter (1993), Initial results of a long-term coral reef monitoring program: Impact of Hurricane Hugo at Buck Island Reef National Monument, St. Croix, U.S. Virgin Islands, *J. Exp. Mar. Biol. Ecol.*, *172*, 171–183, doi:10.1016/0022-0981(93)90096-7.

Bythell, J. C., Z. M. Hillis-Starr, and C. S. Rogers (2000), Local variability but landscape stability in coral reef communities following repeated hurricane impacts, *Mar. Ecol. Prog. Ser.*, *204*, 93–100, doi:10.3354/meps204093.

Connell, J. H. (1978), Diversity in tropical rain forests and coral reefs, *Science*, *199*(4335), 1302–1310, doi:10.1126/science.199.4335.1302.

Cooper, L. (1937), On the ratio of nitrogen to phosphorus in the sea, *J. Mar. Biol. Assoc. U. K.*, *22*(01), 177–182, doi:10.1017/S0025315400011930.

Dollar, S. J., and G. W. Tribble (1993), Recurrent storm disturbance and recovery: A long-term study of coral communities in Hawaii, *Coral Reefs*, *12*(3–4), 223–233, doi:10.1007/BF00334481.

Edmunds, P. J., and J. F. Bruno (1996), The importance of sampling scale in ecology: Kilometer-wide variation in coral reef communities, *Mar. Ecol. Prog. Ser.*, *143*(1–3), 165–171, doi:10.3354/meps143165.

Edmunds, P. J., and J. D. Witman (1991), Effect of Hurricane Hugo on the primary framework of a reef along the south shore of St John, US Virgin Islands, *Mar. Ecol. Prog. Ser.*, *78*(2), 201–204, doi:10.3354/meps078201.

Emanuel, K. (2005), Increasing destructiveness of tropical cyclones over the past 30 years, *Nature*, *436*(7051), 686–688, doi:10.1038/nature03906.

Garcia, H. E., R. A. Locarnini, T. P. Boyer, and J. I. Antonov (2006a), *Nutrients (Phosphate, Nitrate, Silicate), World Ocean Atlas 2005*, vol. 4, 396 pp., NOAA, U.S. Gov. Print. Off., Washington, D. C.

Garcia, H. E., R. A. Locarnini, T. P. Boyer, and J. I. Antonov (2006b), *Dissolved Oxygen, Apparent Oxygen Utilization, and Oxygen Saturation, World Ocean Atlas 2005*, vol. 3, 342 pp., NOAA, U.S. Gov. Print. Off., Washington, D. C.

Gardner, T. A., I. M. Cote, J. A. Gill, A. Grant, and A. R. Watkinson (2005), Hurricanes and Caribbean coral reefs: Impacts, recovery patterns, and role in long-term decline, *Ecology*, *86*(1), 174–184, doi:10.1890/04-0141.

Gaston, K. J. (2000), Global patterns in biodiversity, *Nature*, *405*(6783), 220–227, doi:10.1038/35012228.

Glynn, P. W. (1996), Coral reef bleaching: Facts, hypotheses and implications, *Global Change Biol.*, *2*(6), 495–509, doi:10.1111/j.1365-2486.1996.tb00063.x.

Glynn, P. W., and J. S. Ault (2000), A biogeographic analysis and review of the far eastern Pacific coral reef region, *Coral Reefs*, *19*(1), 1–23, doi:10.1007/s003380050220.

Glynn, P. W., and M. W. Colgan (1992), Sporadic disturbances in fluctuating coral-reef environments: El Nino and coral-reef development in the Eastern Pacific, *Am. Zool.*, *32*(6), 707–718.

Graham, N. A. J., et al. (2008), Climate warming, marine protected areas and the ocean-scale integrity of coral reef ecosystems, *PLoS ONE*, *3*(8), doi:10.1371/journal.pone.0003039.

Harmelin-Vivien, M. L., and P. Laboute (1986), Catastrophic impact of hurricanes on atoll outer reef slopes in the Tuamotu (French Polynesia), *Coral Reefs*, *5*(2), 55–62, doi:10.1007/BF00270353.

Hoegh-Guldberg, O. (1999), Climate change, coral bleaching and the future of the world's coral reefs, *Mar. Freshwater Res.*, *50*(8), 839–866, doi:10.1071/MF99078.

Hoegh-Guldberg, O. (2009), Climate change and coral reefs: Trojan horse or false prophecy?, *Coral Reefs*, *28*(3), 569–575, doi:10.1007/s00338-009-0508-6.

Hoegh-Guldberg, O. (2011), The impact of climate change on coral reef ecosystems, in *Coral Reefs: An Ecosystem in Transition*, edited by Z. Dubinsky and N. Stambler, pp. 391–403, Springer, Dordrecht, Netherlands, doi:10.1007/978-94-007-0114-4_22.

Hoegh-Guldberg, O., et al. (2007), Coral reef under rapid climate change and ocean acidification, *Science*, *318*, 1737–1742, doi:10.1126/science.1152509.

Johannes, R. E., W. J. Wiebe, C. J. Crossland, D. W. Rimmer, and S. V. Smith (1983), Latitudinal limits of coral reef growth, *Mar. Ecol. Prog. Ser.*, *11*(2), 105–111, doi:10.3354/meps011105.

Key, R. M., A. Kozyr, C. Sabine, K. Lee, R. Wanninkhof, J. Bullister, R. Feely, F. Millero, C. Mordy, and T. Peng (2004), A global ocean carbon climatology: Results from GLODAP, *Global Biogeochem. Cycles*, *18*, GB4031, doi:10.1029/2004GB002247.

Kleypas, J. A., J. W. McManus, and L. A. B. Menez (1999), Environmental limits to coral reef development: Where do we draw the line?, *Am. Zool.*, *39*(1), 146–159.

Lewis, J. M., P. M. Hull, K. Q. Weinberger, and L. K. Saul (2008), Mapping uncharted waters: Exploratory analysis, visualization, and clustering of oceanographic data, paper presented at Fourth International Conference on Machine Learning and Applications, Inst. of Electr. and Electron. Eng., San Diego, Calif.

Locarnini, R. A., A. V. Mishonov, J. I. Antonov, T. P. Boyer, and H. E. Garcia (2006), *Temperature, World Ocean Atlas 2005*, vol. 1, 182 pp., NOAA, U.S. Gov. Print. Off., Washington, D. C.

Muscatine, L., and J. W. Porter (1977), Reef Corals: Mutualistic symbioses adapted to nutrient-poor environments, *BioScience*, *27*(7), 454–460, doi:10.2307/1297526.

Pickett, S., and P. White (1985), *The Ecology of Natural Disturbance and Patch Dynamics*, Academic, San Diego, Calif.

Rogers, C. S. (1993), Hurricanes and coral reefs–The intermediate disturbance hypothesis revisited, *Coral Reefs*, *12*(3–4), 127–137, doi:10.1007/BF00334471.

Rosen, B. R. (1988), Progress, problems and patterns in the biogeography of reef corals and other tropical marine organisms (in German), *Helgol. Meersunters.*, *42*(2), 269–301, doi:10.1007/BF02366046.

Salvat, B. (2002), Status of southeast and central Pacific coral reefs "Polynesia Mana node": Cook Islands, French Polynesia, Kiribati, Niue, Tokelau, Tonga, Wallis and Futuna, in *Status of Coral Reefs of the World: 2002*, edited by B. Salvat, pp. 203–215, Aust. Inst. of Mar. Sci., Townsville, Queensland, Australia.

Sandin, S. A., et al. (2008), Baselines and degradation of coral reefs in the Northern Line Islands, *PLoS ONE*, *3*(2), doi:10.1371/journal.pone.0001548.

Sarmiento, J. L., et al. (2004), Response of ocean ecosystems to climate warming, *Global Biogeochem. Cycles*, *18*, GB3003, doi:10.1029/2003GB002134.

Selig, E. R., K. S. Casey, and J. F. Bruno (2010), New insights into global patterns of ocean temperature anomalies: Implications for coral reef health and management, *Global Ecol. Biogeogr.*, *19*(3), 397–411, doi:10.1111/j.1466-8238.2009.00522.x.

Spalding, M. D., C. Ravilious, and E. P. Green (2001), *World Atlas of Coral Reefs*, UNEP World Conserv. Monit. Cent., Univ. of Calif. Press, Berkeley.

Stoddart, D. R. (1969), Ecology and morphology of recent coral reefs, *Biol. Rev. Camb. Philos. Soc.*, *44*(4), 433–498, doi:10.1111/j.1469-185X.1969.tb00609.x.

Szmant, A. M. (2002), Nutrient enrichment on coral reefs: Is it a major cause of coral reef decline?, *Estuaries Coasts*, *25*(4), 743–766, doi:10.1007/BF02804903.

Veron, J., L. Devantier, E. Turak, A. Green, S. Kininmonth, M. Stafford-Smith, and N. Peterson (2009), Delineating the coral triangle (in Japanese), *Galaxea. J. Coral Reef Stud.*, *11*, 1–6, doi:10.3755/jcrs.11.1.

Waddell, J. E., and A. M. Clark (2008), The state of coral reef ecosystems of the United States and Pacific freely associated states: 2008, report, 569 pp., NOAA/NCCOS Cent. for Coastal Monit. and Assess., Silver Spring, Md.

Wilkinson, C. (2008), *Status of Coral Reefs of the World, Rep.*, 296 pp., Global Coral Reef Monit. Network and Reef and Rainforest Res. Cent., Townsville, Queensland, Australia.

Willig, M. R., D. M. Kaufman, and R. D. Stevens (2003), Latitudinal gradients of biodiversity: Pattern, process, scale, and synthesis, *Annu. Rev. Ecol. Evol. Syst.*, *34*, 273–309, doi:10.1146/annurev.ecolsys.34.012103.144032.

Woodley, J. D., et al. (1981), Hurricane Allen's impact on Jamaican coral reefs, *Science*, *214*(4522), 749–755, doi:10.1126/science.214.4522.749.

Wyrtki, K. (1975), El Nino: Dynamic response of equatorial Pacific Ocean to atmospheric forcing, *J. Phys. Oceanogr.*, *5*(4), 572–584, doi:10.1175/1520-0485(1975)005<0572:ENTDRO>2.0.CO;2.

Zhang, C. D. (1996), Atmospheric intraseasonal variability at the surface in the tropical western Pacific Ocean, *J. Atmos. Sci.*, *53*(5), 739–758, doi:10.1175/1520-0469(1996)053≤0739:AIVATS≥2.0.CO;2.

Zhang, C. D. (2005), Madden-Julian oscillation, *Rev. Geophys.*, *43*, RG2003, doi:10.1029/2004RG000158.

L. A. Freeman, A. J. Miller, R. D. Norris, and J. E. Smith, Scripps Institution of Oceanography, University of California, San Diego, La Jolla, CA 92093-0224, USA. (lfranck@ucsd.edu)
